# PROS1‐MERTK Axis Drives Tumor Microenvironment Crosstalk and Progression in Papillary Thyroid Microcarcinoma

**DOI:** 10.1002/advs.202413474

**Published:** 2025-05-28

**Authors:** Wenqian Zhang, Ye Zhang, Zhu Liu, Zhiyuan Wang, Huaqin Wang, Xiaoyu Ji, Hongyue Su, Fan Yang, Lirong Yan, Ying Xu, Hao Zhang, Wei Sun

**Affiliations:** ^1^ Department of Head and Neck Surgery Cancer Hospital of China Medical University Liaoning Cancer Hospital Shenyang 110042 China; ^2^ The First Laboratory of Cancer Institute The First Hospital of China Medical University Shenyang 110001 China; ^3^ Department of Thyroid Surgery The First Hospital of China Medical University Shenyang 110001 China; ^4^ Department of Biochemistry & Molecular Biology China Medical University Shenyang 110122 China; ^5^ School of Medicine Southern University of Science and Technology Shenzhen 518055 China

**Keywords:** papillary thyroid microcarcinoma, single‐cell RNA sequencing, tumor microenvironment

## Abstract

The incidence of papillary thyroid carcinoma (PTC) has been rising annually, with papillary thyroid microcarcinoma (PTMC) accounting for more than half of the cases. While most PTMCs exhibit indolent growth and a favorable prognosis, some undergo clinical progression with poor outcomes. Thus, identifying biomarkers associated with PTC, particularly those related to PTMC progression, is crucial for precise risk stratification and treatment planning. This study utilized single‐cell RNA sequencing on 19 surgical tissue specimens from 15 patients, including four para‐tumor tissues, four non‐progressive PTMCs, five progressive PTMCs, and six progressive PTCs. Key findings are corroborated through in vivo and in vitro experiments. Single‐cell RNA sequencing and spatial transcriptomics characterized the cellular ecosystem within PTC, revealing multi‐directional evolutionary patterns as PTMC progresses. Analysis of progression‐specific alterations in intercellular communication networks highlighted the PROS1‐MERTK signaling interaction as pivotal in PTMC progression. In vitro and in vivo models confirm that the PROS1‐MERTK axis accelerates PTMC progression via paracrine and autocrine signaling. Furthermore, NFYB and FOXP2 are identified as activators of PROS1 transcription in fibroblasts, promoting PTMC progression through the MERTK/WNT/TGF‐β signaling. These findings underscore the PROS1/MERTK axis as a critical component of the cellular microenvironment and a key regulatory mechanism in PTMC progression.

## Graphical abstract

Identifying biomarkers associated with PTC, particularly those related to PTMC progression, is crucial for precise risk stratification and treatment planning. This study utilized single‐cell RNA sequencing on 19 surgical tissue specimens, confirmed PROS1/MERTK axis as a critical component of the cellular microenvironment and a key regulatory mechanism in PTMC progression.

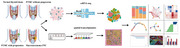

## 1 Introduction

Thyroid cancer, the most prevalent endocrine malignancy, encompasses three types: differentiated (papillary and follicular carcinoma), undifferentiated (poorly differentiated and anaplastic carcinoma), and medullary carcinoma. Among these, papillary thyroid carcinoma (PTC) is the most common, accounting for over 90% of thyroid cancer cases.^[^ ^1^ ^]^ The use of high‐resolution ultrasound has increased the detection rate of papillary thyroid microcarcinoma (PTMC), defined as PTC with a maximum diameter of ≤1 cm, which significantly contributes to the rising global incidence of PTC.^[^ ^2^ ^]^ Several signaling pathways, such as MAPK/ERK and PI3K/AKT, are implicated in thyroid cancer pathogenesis; however, the mechanisms driving PTC progression, particularly in PTMC, remain poorly understood. While most PTMCs exhibit slow growth and a favorable prognosis, with 10‐year survival rates ranging from 93.5% to 97% following standard treatment,^[^ ^1^, ^3^, ^4^ ^]^ a shift in clinical perspective occurred in 2013 with the introduction of the concept of “overdiagnosis and overtreatment” of PTMC by the Mayo Clinic in the British Medical Journal.^[^ ^5^ ^]^ This has led to the adoption of active surveillance (AS) as an alternative to immediate surgery for nonprogressive PTMC by hospitals in Japan, the United States, Korea, and other countries. However, not all PTMCs follow an indolent course; some exhibit rapid clinical progression, including tumor enlargement, lymph node metastasis (LNM), and extrathyroidal extension (ETE) in the short term. Clinical progression occurs in 0.4%–28.8% of patients under AS, with 1.3%–2‐8.8% experiencing tumor enlargement, 0.9%–3.9% developing LNM, and 0.4%–1.3% presenting with ETE.^[^ ^6^ ^]^ Therefore, identifying biomarkers for PTC, particularly for PTMC progression, is critical for accurate risk stratification and informed treatment decision‐making.

The tumor microenvironment consists of a variety of cell types, including malignant, stromal, and immune cells. Its dynamic and heterogeneous nature, along with tissue‐specific attributes, forms a complex, multi‐layered regulatory network that governs cell interactions during malignant transformation.^[^ ^7^, ^8^ ^]^ These intercellular communication networks are essential in tumorigenesis, progression, treatment resistance, immune infiltration, and inflammation.^[^ ^9^, ^10^, ^11^ ^]^ Substantial evidence links tumor microenvironment interactions with PTC progression. For example, Kucuk et al. reviewed pathological sections of 163 PTMCs and found correlations between mast cell infiltration and tumor invasion, neutrophil infiltration and capsular invasion, and plasma cell infiltration with larger tumor size.^[^ ^12^ ^]^ Likewise, Zhang et al. analyzed pathological sections of 406 PTCs, conducting immune infiltration scoring and identifying the immune infiltration score as an independent risk factor for central LNM in PTC.^[^ ^13^ ^]^ Recently, single‐cell RNA sequencing (scRNA‐Seq) has been employed to investigate ligand‐receptor interactions across all cell types in the microenvironment, offering an effective method to explore tumor microenvironment heterogeneity and intercellular communication networks.^[^ ^14^, ^15^ ^]^ Despite these advancements, the mechanisms through which tumor cells modulate microenvironmental cells and the influence of the cellular microenvironment on PTMC progression remain unclear.

TAM receptors, comprising TYRO3, AXL, and MERTK, are a family of type I receptor tyrosine kinases (RTKs). Their endogenous ligands, Protein S (PROS1) and growth arrest‐specific gene 6 (GAS6) mediate their activation. These receptors are frequently overexpressed in various cancers, where they activate signaling pathways that promote carcinogenesis and cell survival, invasion, metastasis, chemotherapy resistance, tumor grading, and poor prognosis.^[^ ^16^, ^17^, ^18^, ^19^, ^20^, ^21^, ^22^ ^]^ In addition to their expression in tumor cells, TAM receptors are also present in infiltrating myeloid cells within the tumor microenvironment, where they act similarly to immune checkpoint inhibitors by suppressing the host's anti‐tumor immune response.^[^ ^23^, ^24^, ^25^, ^26^ ^]^ Despite their significant roles in cancer biology, research on TAM receptor signaling in thyroid carcinoma is limited. Notably, AXL is highly expressed in undifferentiated, medullary, and papillary thyroid carcinomas.^[^ ^27^ ^]^ PROS1 expression is significantly correlated with lymph node staging in patients with PTC, and downregulation of PROS1 inhibits PTC cell proliferation and migration.^[^ ^28^ ^]^ While these findings underscore the relevance of TAM receptor signaling in PTC, many questions remain unanswered, particularly regarding the full extent of TAM receptor signaling in the PTMC tumor microenvironment.

This study employed scRNA‐Seq, spatial transcriptomics, multiple immunofluorescence assays, and both in vivo and in vitro validation to comprehensively investigate the mechanisms of progression within the PTMC tumor microenvironment. The samples analyzed included four para‐tumoral (normal) tissues, four non‐progressive PTMCs (from patients who underwent five years of active surveillance without clinical progression), five progressive PTMCs, and six progressive PTC tissues. The goal was to characterize the composition, cell‐cell interactions, and heterogeneity of the tumor microenvironment at various stages of progression. Key interactions between cancer‐associated fibroblasts (CAFs), tumor cells expressing PROS1, and tumor cells expressing MERTK were identified as central drivers of PTMC progression. This investigation into PROS1‐MERTK receptor‐mediated signaling pathways provides new insights for the clinical management and early detection of PTMC progression.

## 2 Results

### 2.1 Defining the Cellular Ecosystem of PTC Through Single‐Cell Transcriptional Profiling

**Figure 1:**
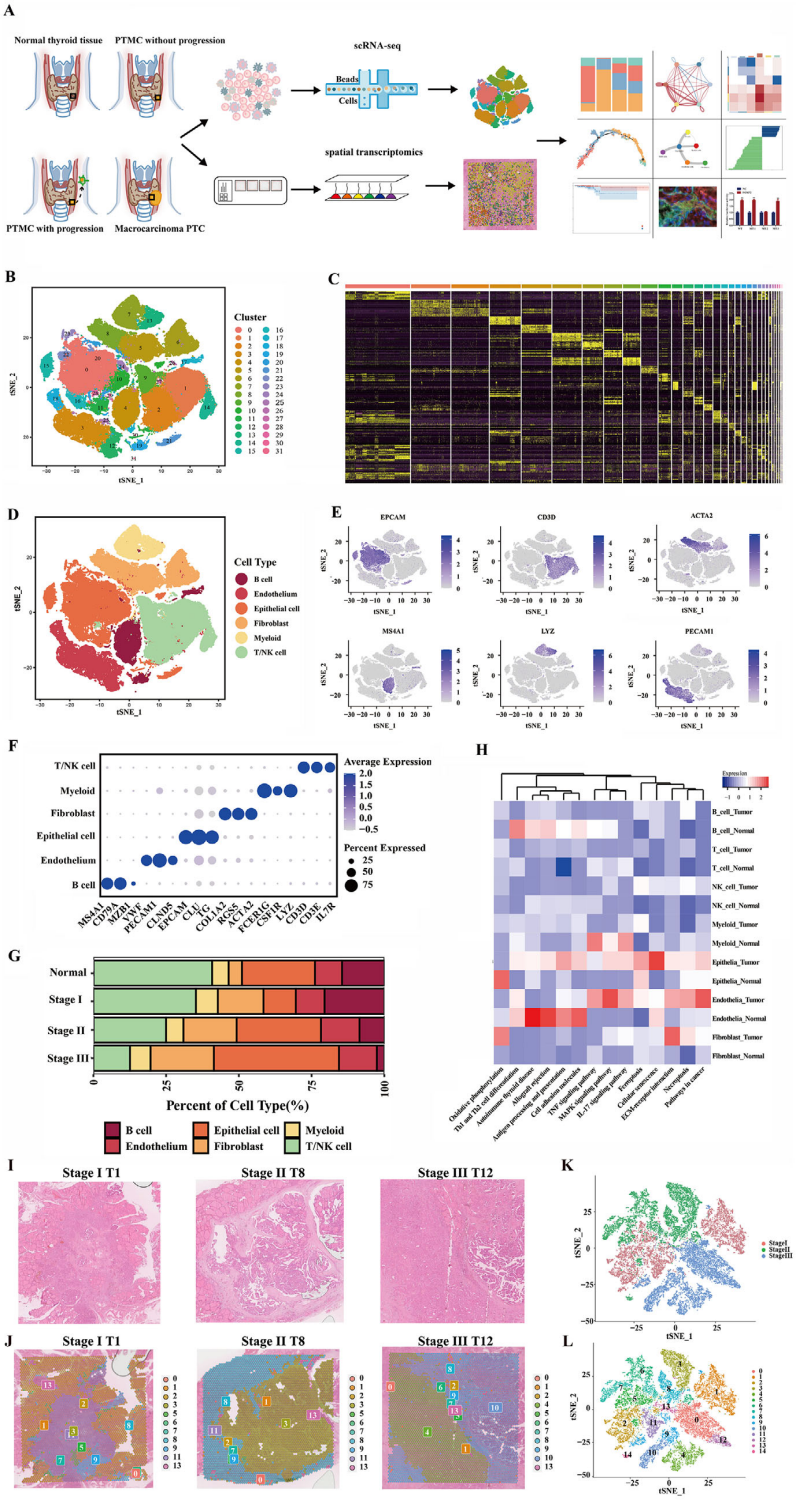
Characterization of the tumor microenvironment in different thyroid samples through single‐cell transcriptomic sequencing. A) Overview of the study design and workflow. Single‐cell suspensions were collected from four normal, four non‐progressive PTMC, five progressive PTMC, and six progressive PTC tissues, followed by single‐cell transcriptomic sequencing using the 10× Genomics platform. Paraffin‐embedded tissue sections were utilized for spatial transcriptomics. B) t‐SNE plot displaying 146529 single cells profiled in this study, color‐coded by clusters. C) Heatmap illustrating the expression levels of differentially expressed genes across 32 clusters. D) t‐SNE plot of single cells, color‐coded by major cell type. E) Feature plots showing the expression of canonical marker genes for distinct cell types. F) Dot plot representing the expression of canonical marker genes across all major cell types. G) Bar plots illustrating the proportion of major cell lineages at each stage. H) Heatmap showing the differentially activated pathways in PTC versus non‐malignant groups. I) H&E staining of representative tissue sections. J) Spatial distribution of different clusters within tumor (and adjacent) tissues on 10× Visium capture slides. The spots are also summarized and labeled according to different stage type specimens K) and clusters L).

In this study, scRNA‐Seq was performed to characterize the cellular components within the PTC microenvironment (**Figure** **1**A). Following the exclusion of low‐quality cells, principal component analysis classified 28 377, 28 284, 40 788, and 49 080 cells from normal, stage I, stage II, and stage III tissues, respectively, into 32 distinct clusters. On average, each cell contained ≈3000 genes and 10 000 UMIs (Figure 1B, Figure S1A–F). The heatmap in Figure 1C presents the differential gene expression profiles for each cluster. Based on the expression of prototypical cell type‐specific marker genes, cells were categorized into six primary cell types: thyrocytes (EPCAM), fibroblasts (ACTA2), myeloid cells (LYZ), endothelial cells (PECAM1), T and NK cells (CD3D), and B cells (MS4A1; Figure 1D–F). The proportions of these cell types varied across patient samples (Figure S1G). Disease progression was associated with a gradual decline in T and NK cells, along with an increase in fibroblasts (Figure 1G). Moreover, the proportion of B cells decreased in stages II and III compared to normal or stage I samples (Figure 1G). The most pronounced changes were observed in epithelial cells (Figure 1G). The heatmap in Figure 1H illustrates the differential enrichment of cancer hallmark genes across cell clusters from normal and tumor tissues. Epithelial cells from normal tissues were highly enriched in oxidative phosphorylation, whereas tumor epithelial cells exhibited enrichment in cellular senescence, ferroptosis, antigen processing and presentation, pathways in cancer, and ECM‐receptor interactions (Figure 1H). Endothelial cells from normal tissues were enriched in autoimmune thyroid disease, allograft rejection, cell adhesion molecules, and antigen processing and presentation, while tumor endothelial cells showed significant enrichment in the MAPK signaling pathway, pathways in cancer, and ECM‐receptor interactions (Figure 1H).

To further investigate changes in the tumor microenvironment during PTC progression, spatial transcriptome sequencing was conducted on PTC tissues from three stages of progression obtained from the same patients who underwent single‐cell sequencing. Hematoxylin and eosin (H&E) staining was performed (Figure 1I, Figure S1H), and the spots captured by 10x Visium from all six specimens were divided into 15 clusters, aligned with the H&E images. These clusters exhibited significant heterogeneity within the specimens. In stages I, II, and III, a clear boundary was observed between the tumor and adjacent tissues. Stage I and II tumor regions showed relatively uniform consistency, while stage III tumor regions were notably divided into two distinct categories, indicating substantial heterogeneity in advanced PTC tumors (Figure 1J–l, Figure S1H).

### 2.2 Identification and Characterization of Cancer Cell States in PTC

**Figure 2:**
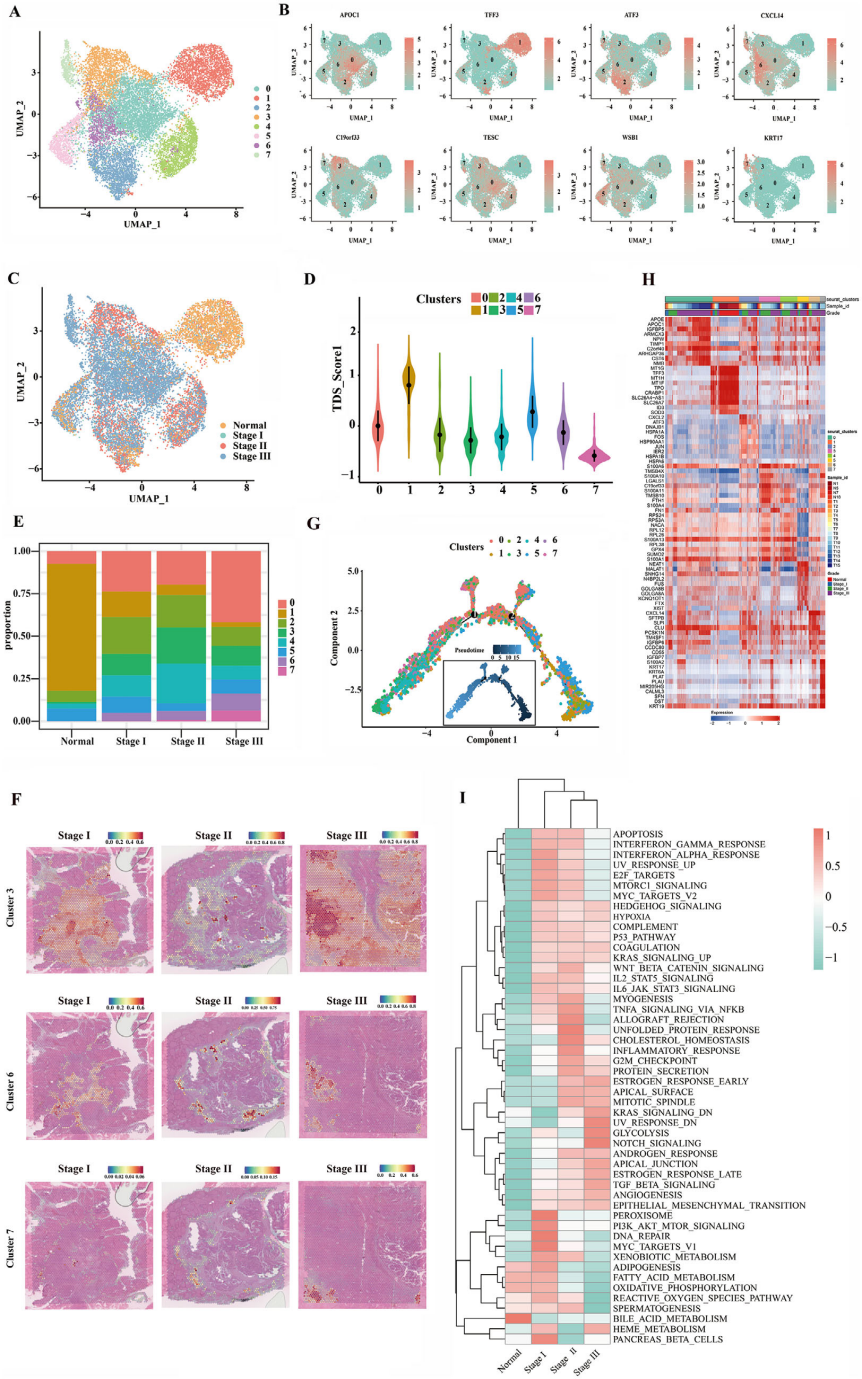
Identification of thyrocyte heterogeneity between paracancerous tissues and tumors at varying progression levels. A) UMAP plot depicting distinct clusters based on gene expression differences for 14965 thyrocytes that passed quality control. B) Feature plots displaying the normalized expression of highly expressed genes in each thyrocyte subcluster. C) UMAP projection visualizing the distribution of thyrocytes across tissues, color‐coded by tissue type. D) Violin plots showing the TDS score distribution for each thyrocyte cluster. E) Bar plots indicating the proportion of eight thyrocyte subclusters across each stage. F) Mapping of the three thyrocyte clusters identified through single‐cell sequencing onto tissue slices. G) Monocle pseudotime trajectory analysis of thyrocytes using highly variable genes, with each dot representing a single cell, colored according to its cluster label. H) Heatmap displaying the top 10 differentially expressed genes across the eight thyrocyte clusters. I) Heatmap showing the 50 hallmark pathways (rows) significantly enriched in thyrocytes for each stage (columns).

Graph‐based clustering of the epithelial cell population using Seurat identified eight clusters (C0–C7) (**Figure** **2**A). Most epithelial cells from normal tissues were concentrated in cluster C1, whereas cancer cells exhibited patient‐specific expression patterns (Figure 2B,C). The thyroid differentiation score (TDS) revealed that cluster C1 exhibited the highest degree of differentiation (Figure 2D), consistent with its predominance in normal tissues (Figure 2E).^[^ ^29^ ^]^ As the disease progressed, the proportion of C1 cells significantly decreased, from an average of 74% in normal tissues to 14% in stage I, 6% in stage II, and 2% in stage III, indicating a process of dedifferentiation (Figure 2E). In contrast, clusters C0, C2, C3, C6, and C7 exhibited significantly increased proportions in tumor tissues, particularly in stage III samples, with a smaller portion originating from stage II (Figure 2D,E). C3, C6, and C7, which were primarily derived from tumor tissues, were designated as tumor‐associated epithelial cells. Spatial transcriptome results corroborated these findings, as mapping C3, C6, and C7 onto the spatial slices showed significant distribution in the tumor area compared to the adjacent non‐cancerous area. C3 had the largest distribution, while C6 and C7 were more localized. As PTC progressed, the proportions of C6 and C7 gradually increased, especially C7, which showed a marked increase in abundance in stage III. Notably, the lower left corner of the stage III slice, composed mainly of interstitial tissue with some local satellite foci, demonstrated significant co‐localization of these foci with C7 tumor cells, indicating a close association between C7 and PTC progression (Figure 2F, Figure S1I). Pseudotime trajectory analysis confirmed that clusters C3, C6, and C7 predominantly represented cells at advanced pseudotime stages, while C1 was primarily composed of early‐stage cells, suggesting a gradual transition from normal epithelial cells to highly malignant tumor cells (Figure 2G). The top DEGs in each subcluster were visualized through a heatmap (Figure 2H). KEGG‐based functional enrichment analysis revealed that DEGs in C1 were primarily associated with normal metabolic pathways, while DEGs in tumor‐derived epithelial cells were enriched in various cancer‐related pathways and functions involved in external interactions (Figure S2A–H). GSVA further confirmed the upregulation of angiogenesis, Hedgehog signaling, Kras signaling, inflammation (including inflammatory response and IL6‐JAK‐STAT3 signaling), Wnt‐β catenin signaling, and protein secretion in tumor‐derived epithelial cells (Figure 2I, Figure S1J). These results suggest that tumor‐derived epithelial cells exhibit more active biological processes and stronger interactions with the tumor microenvironment. In contrast, C1 exhibited high activity in metabolic pathways related to lipid metabolism (including fatty acid metabolism, bile acid metabolism, and adipogenesis), oxidative stress, and the unfolded protein response (Figure S1J), aligning with the biological function of thyroid hormone production in thyrocytes. Conversely, fatty acid metabolism was downregulated in C6 and C7 (Figure S1J), further confirming their dedifferentiation. Similar pathways were observed in stages II and III samples, with distinct differences such as notable upregulation of the inflammatory response in stage II and significant upregulation of glycolysis and the NOTCH signaling pathway in stage III (Figure 2I).

### 2.3 Enhancement of PROS1‐MERTK Mediated Fibroblast‐Tumor Epithelial Cell Communication in PTMC with Progression

**Figure 3:**
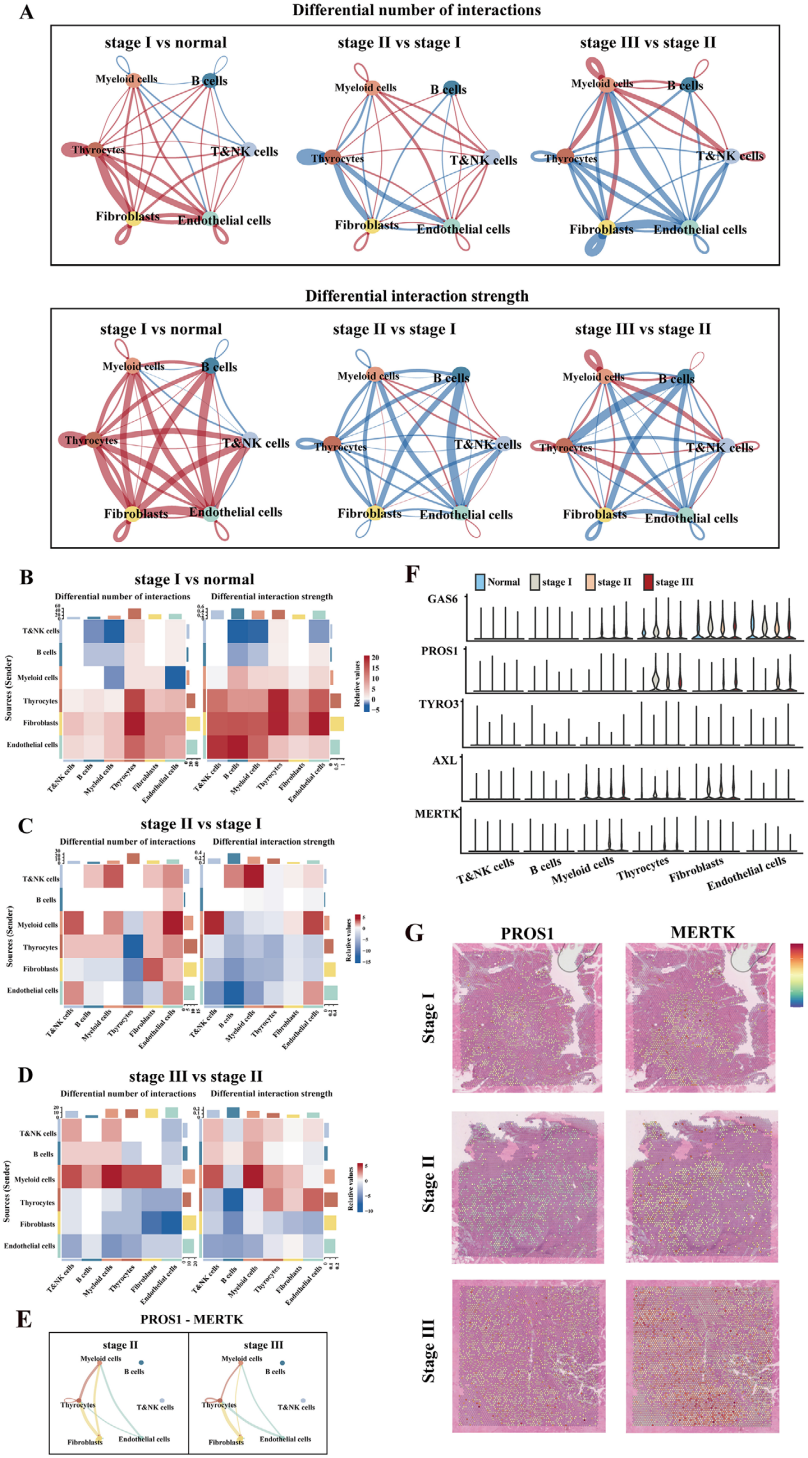
Global alterations in the intercellular communication network during PTC progression. A) Schematic diagram illustrating cell–cell communication between major cell types. Circle plots depicting the interaction quantity and strength between major cell types. Blue lines represent decreased communication, while red lines indicate increased communication. B–D) Heatmaps showing interaction quantity (left) and interaction strength (right) between major cell types, with blue lines indicating decreased communication and red lines indicating increased communication in the former compared to the latter. E) Circle plots depicting the interaction between PROS1‐MERTK ligand‐receptor pairs across major cell types in tumors at different stages. F) Violin plots illustrating the expression of TAM signaling‐related genes across each major cell type. G) Expression of PROS1 and MERTK on spatial tissue slices.

Considering the active cellular activity in tumor‐derived epithelial cells, scRNA‐Seq data were integrated with the ligand‐receptor interaction database to construct an intercellular signaling pathway atlas of the microenvironment. Signaling pathways were compared between stage I and normal tissues, stages II and I, and stages III and II, respectively. The number and strength of interactions among epithelial cells, fibroblasts, and endothelial cells generally increased in stage I compared to normal controls (**Figure** **3**A,B). However, interactions between fibroblasts and endothelial cells decreased in stages II (Figure 3A,C) and III (Figure 3A,D).

Quantification of information flows for each signaling pathway identified PROS1, INF‐II, and POSTN signaling networks, with distinct trends of normal < stage I < stage II ≤ stage III or normal > stage I > stage II ≥ stage III (Figure 3E,F; Figure S2I–N). Analysis of scRNA‐Seq data revealed that the TAM signaling ligand PROS1 was specifically expressed in tumor‐associated epithelial cells, CAFs, and endothelial cells in stages II and III (Figure 3F, Figure S2O). The interaction between epithelial cells and fibroblasts was stronger than that of other cell types (Figure 3E). Additionally, GAS6 was widely expressed in myeloid cells, fibroblasts, endothelial cells, thyroid epithelium, and tumor cells (Figure 3F). TYRO3 expression was rarely detected, while AXL was expressed in myeloid cells, tumor epithelial cells, and CAFs. MERTK was primarily expressed in myeloid cells and tumor epithelial cells in stages II and III tissues (Figure 3F, Figure S2O). Moreover, MERTK expression was detected in tumor‐associated macrophages, with significant upregulation observed in stages II and III tissues(Figure S2P). High MERTK expression in tumor‐associated macrophages correlated with multiple cancer‐related pathways, as indicated by KEGG and GO analyses (Figure S2Q).

Examination of individual TAM receptor‐mediated signaling pathways revealed that the GAS6‐AXL pathway was prevalent in both normal and tumor tissues, albeit with distinct intercellular communication patterns (Figure S2I). In normal tissues, GAS6 signaling involved thyrocytes, fibroblasts, and endothelial cells, with myeloid cells as exclusive recipients (Figure S2I,J). In tumor tissues, GAS6‐AXL‐mediated communication was more complex, involving myeloid cells, fibroblasts, endothelial cells, and occasionally thyrocytes (Figure S2I). PROS1‐AXL communication was tumor‐specific and absent in normal tissues (Figure S2J). In stages II and III, fibroblasts acted as both senders and receivers of PROS1 signals (Figure 3E, Figure S2J). The spatial expression and distribution of PROS1 and MERTK on H&E pathological sections aligned with single‐cell analysis, showing their primary distribution in tumor areas and increased expression as PTC progressed (Figure 3G, Figure S2R). In the IFN‐II signaling pathway network, IFNG, a specific signaling molecule produced by T and NK cells in stages II and III tissues, facilitated communication among T and NK cells, myeloid cells, fibroblasts, thyrocytes, and endothelial cells (Figure S2K,L). In contrast, POSTN acted as an autocrine signal, predominantly produced by CAFs in stages II and III tissues (Figure S2M,N).

### 2.4 Release of PROS1 by Adipogenic CAFs in PTMC with Progression

**Figure 4:**
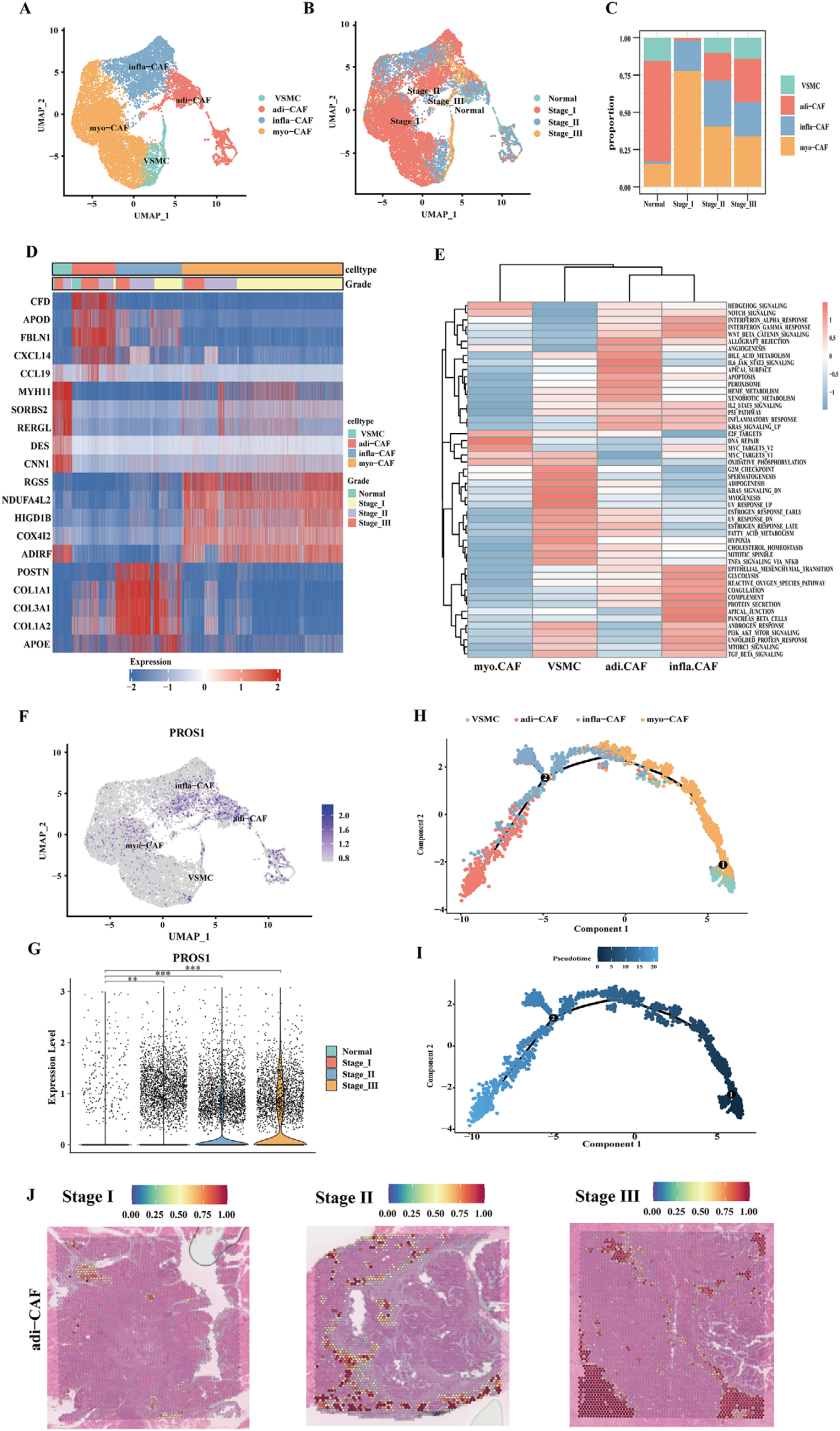
Diversity of cancer‐associated fibroblasts (CAFs) in the tumor microenvironment. A) UMAP plot displaying clusters, differentiated by colors, showing four distinct fibroblast subtypes based on gene expression differences in 14705 fibroblasts. B) UMAP plot illustrating the fibroblast landscape, color‐coded by tumor stage. C) Bar plots representing the proportion of each fibroblast subtype across different stages. D) Heatmap showing the top five highly expressed genes in each fibroblast subtype. E) Heatmap displaying the 50 significantly enriched hallmark pathways (rows) for fibroblasts across each cluster (columns). F) Feature plot indicating the normalized expression of PROS1 in each fibroblast subtype. G) Violin plots demonstrating the expression of PROS1 across different stages. H‐I) Monocle pseudotime trajectory analysis of fibroblasts using highly variable genes. Each dot on the pseudotime curve represents a single cell, colored according to its cluster label. J) Mapping of adi‐CAFs identified by single‐cell sequencing onto tissue slices. All *P*‐values were calculated using an unpaired two‐sided Wilcoxon rank‐sum test. **, *P* < 0.01; ***, *P* < 0.001.

The POSTN and INF‐II signals were exclusively produced by fibroblasts, T cells, and NK cells, while PROS1 expression originated from epithelial cells, fibroblasts, and endothelial cells. Considering the critical role of tumor cells in the microenvironment and the dominant contribution of fibroblasts to PROS1 signaling, further principal component analysis (PCA) was conducted on fibroblast populations. Fibroblasts were classified into four subpopulations based on the genes with the most variable expression: vascular smooth muscle cells (VSMCs), cancer‐associated myofibroblasts (myo‐CAFs), inflammatory CAFs (infla‐CAFs), and adipogenic CAFs (adi‐CAFs). These subpopulations exhibited notable heterogeneity in their infiltration proportions across the four sample types (**Figure** **4**A–C, Figure S3A,B). In tumor samples, myo‐CAFs represented the highest proportion, which decreased with advancing tumor stages. Adi‐CAFs were notably enriched in stages II and III, while infla‐CAFs were predominantly present in tumors, maintaining consistent proportions across all stages. Heatmap analysis of differential gene expression revealed substantial transcriptional profile variation among the fibroblast subpopulations (Figure 4D). GSVA analysis further indicated the upregulation of pathways linked to E2F targets, MYC targets V1 and V2, DNA repair in myo‐CAFs, and pathways related to the G2M checkpoint, myogenesis, and cholesterol homeostasis in VSMCs (Figure 4E). In contrast, adi‐CAFs and infla‐CAFs showed upregulation of pathways associated with tumor progression, including Hedgehog, PI3K/Akt/mTOR, Wnt/β‐Catenin, and TGF‐β signaling pathways (Figure 4E). Notably, adi‐CAFs highly expressed PROS1 (Figure 4F, Figure S3C), with stage III adi‐CAFs identified as the primary source of PROS1 expression (Figure 4G, Figure S3D). Pseudotime trajectory analysis revealed that adi‐CAFs had the highest pseudotime score, while VSMC and myo‐CAFs were situated in the early pseudotime stages, suggesting that adi‐CAFs may differentiate from VSMCs or myo‐CAFs in response to tumor stimuli (Figure 4H,I, Figure S3E,F). Spatial transcriptomics analysis showed that VSMCs subgroups failed to map onto spatial slices, likely due to their low proportion. The remaining fibroblast subgroups exhibited significant spatial heterogeneity (Figure 4J, Figure S3G–I). Adi‐CAFs were predominantly located in the interstitial regions surrounding the tumor, with infiltration levels increasing as the tumor progressed. Both infla‐CAFs and myo‐CAFs showed a trend of infiltration from the tumor periphery toward the center in stage I, but this trend diminished with tumor progression, and by stage III, myo‐CAFs infiltration was nearly absent.

### 2.5 TAM Receptor‐Mediated Intercellular Communication in PTC

**Figure 5:**
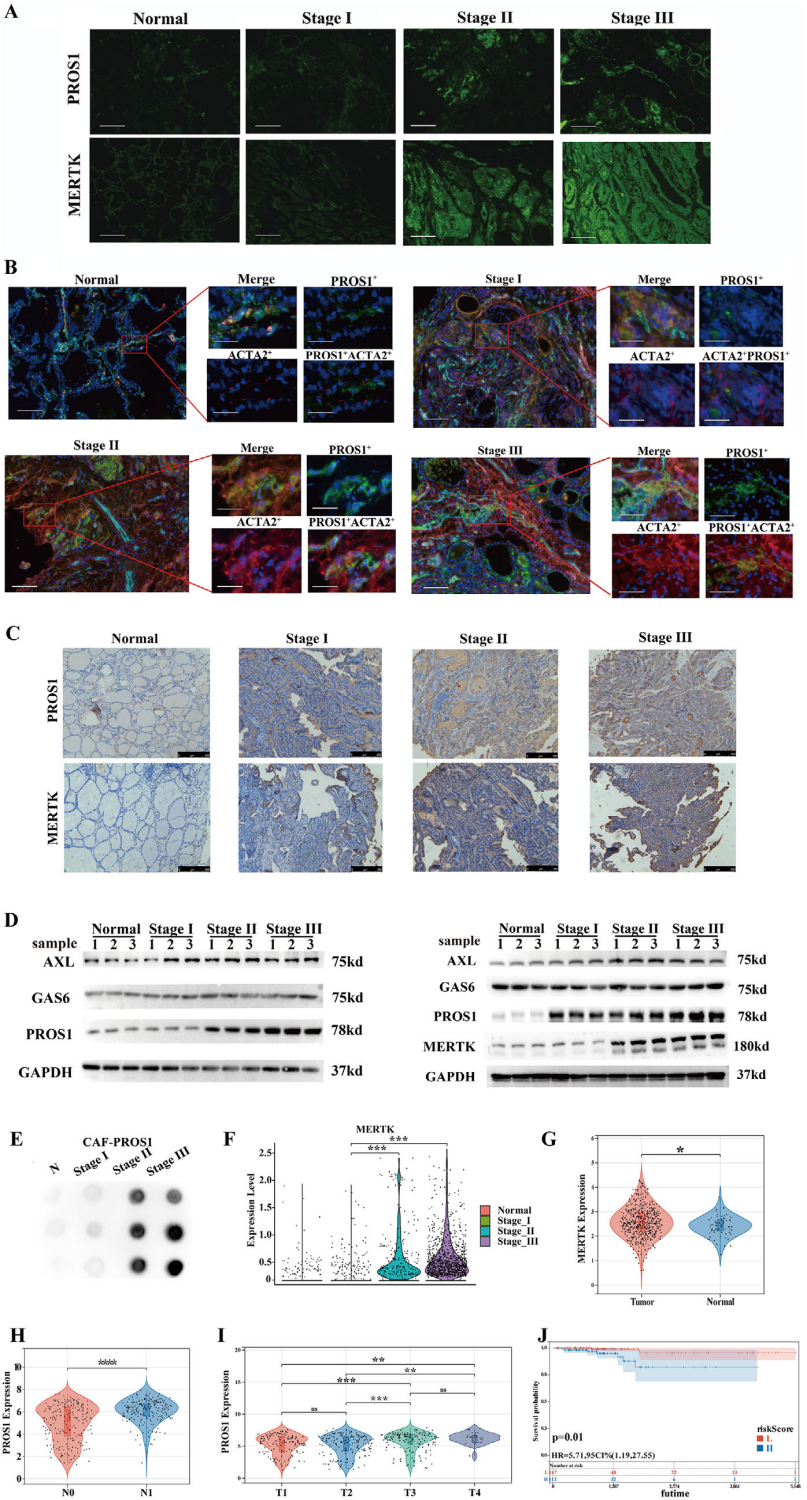
TAM receptor‐mediated intercellular communication in PTC. A) Immunofluorescence staining of PROS1 and MERTK in normal thyroid tissues and PTC samples at different stages (*n =* 10 per group). Scale bars = 50 µm. B) Representative immunofluorescence images showing the co‐localization of PROS1 with ACTA2^+^ fibroblasts in Stage II and III PTC samples (*n =* 10 per group). Inset panels show magnifications of the highlighted regions in red. Scale bars = 50 µm (large panel), 20 µm (small panel). C) Representative IHC staining of PROS1 and MERTK in PTC tissues at various stages and adjacent non‐cancerous tissues (*n =* 10 per group). Scale bar = 250 µm. D) Western blot analysis showing the protein expression levels of TAM signaling‐related genes in primary fibroblast and epithelial cell cultures derived from four different tissue types (*n =* 3). E) Dot blot assay measuring PROS1 expression in the supernatant of primary fibroblast culture medium (*n =* 3). F) Violin plot showing MERTK expression in thyrocytes at different stages. G) Significant overexpression of MERTK in tumors, confirmed using the TCGA database. H‐J) Correlation between PROS1 expression and advanced clinical staging of PTC, confirmed using the TCGA database, indicating that high PROS1 expression correlates with poorer prognosis. Log‐rank test was used to assess differences. All *P*‐values were calculated using an unpaired two‐sided Wilcoxon rank‐sum test. ns, *P* ≥ 0.05; *, *P* < 0.05; **, *P* < 0.01; ***, *P* < 0.001; ****, *P* < 0.0001.

To validate the expression patterns of TAM receptors (MERTK and AXL) and their ligands (GAS6 and PROS1) across various tumor stages, multiple immunofluorescence assays were conducted (**Figure** **5**A, Figure S4A). The GAS6 signal was most prominent in non‐progressive PTMC tissues, with no significant differences observed between invasive PTMC and progressive PTC (Figure S4A). Both PROS1 and MERTK signals were markedly elevated in invasive PTMC and progressive PTC, with MERTK showing particularly high expression (Figure 5A). In progressive PTC (stages II and III), nearly all tumor cells exhibited strong MERTK expression, while AXL expression remained unchanged between normal and tumor tissues (Figure S4A). Immunofluorescence assays, consistent with single‐cell sequencing data, showed no co‐localization of ACTA2+ fibroblasts and PROS1 in normal tissues or non‐progressive PTMC, but co‐localization was evident in invasive PTMC and progressive PTC (Figure 5B).

Immunohistochemistry on 50 samples each from normal tissues, stage I, stage II, and stage III specimens confirmed significantly elevated MERTK expression in stages II and III. PROS1 expression was significantly higher in tumors compared to normal tissues (Figure 5C, Figure S4B). We performed an immunoreactive score analysis on MERTK expression between 50 non‐progressing PTMC (stage I) and 100 clinically progressing PTMC (stage II, stage III). The results revealed that the immunoreactive score for non‐progressing PTMC was significantly lower compared to that of clinically progressing PTMC (Figure S4B). Primary fibroblast cultures revealed significant overexpression of PROS1 in stage II and III CAFs compared to stage I CAFs and NFs (Figure 5D). Similarly, primary PTC cell cultures demonstrated significant PROS1 overexpression in PTC cells relative to normal thyroid follicular epithelial cells (Figure 5D). MERTK expression was also significantly higher in stages II and III PTC cells compared to stage I and normal thyroid follicular epithelial cells (Figure 5D). In contrast, AXL and TYRO3 expression did not differ significantly between normal and tumor tissues (Figure 5D, Figure S4C). Dot blot assays revealed increased PROS1 release in stage II and III CAFs compared to stage I CAFs or NFs (Figure 5E), with no significant difference in GAS6 secretion between NFs and CAFs (Figure S4D).

Single‐cell data further supported the upregulation of MERTK in thyrocytes, particularly in clusters 2, 3, 6, and 7, which are associated with tumor progression (Figure 5F, Figure S4E). TCGA bulk sequencing data (568 samples) confirmed significant upregulation of PROS1 and MERTK in tumors compared to adjacent tissues (Figure 5G, Figure S4F). Subgroup analysis of the TCGA cohort revealed that high PROS1 expression correlated positively with higher T stage and LNM, resulting in a poorer prognosis (Figure 5H–J).

### 2.6 PROS1‐MERTK Promotes PTC Progression Through Paracrine And Autocrine Pathways

**Figure 6:**
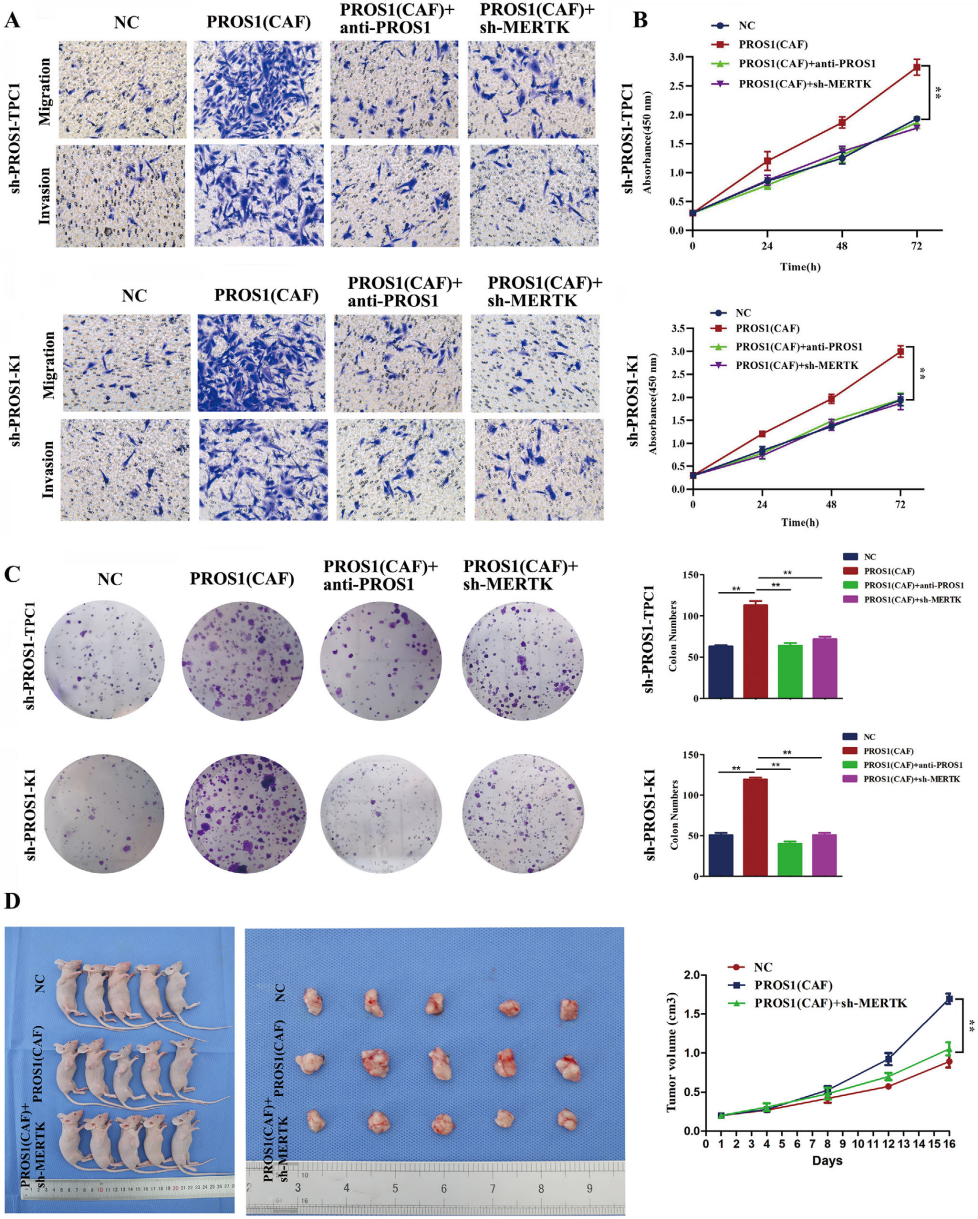
PROS1‐MERTK promotes PTC progression through paracrine and autocrine pathways. A) Transwell assays assessing migration and invasion in PTC cells under various treatments targeting paracrine signaling (*n =* 3). B) CCK‐8 assay evaluating the proliferation of PTC cells subjected to different paracrine treatments (*n =* 3). C) Colony formation assay to assess the proliferative ability of PTC cells after treatments targeting paracrine signaling (*n =* 3). D) Subcutaneous implantation in mouse models inoculated with NC, Lv‐PROS1 CAFs + NC, or Lv‐PROS1 CAFs + sh‐MERTK K1 cells (*n =* 5), with tumor volumes measured in each group. All *P*‐values were calculated using an unpaired two‐sided Student's t‐test. Data are presented as mean ± SD. **, *P* < 0.01.

Cell function assays confirmed the role of the PROS1‐MERTK ligand‐receptor interaction in PTC. The results showed that the supernatant from PROS1‐overexpressing CAFs significantly enhanced migration, invasion, and proliferation in sh‐PROS1 TPC1 and K1 cells. These effects were diminished by treatment with a PROS1‐neutralizing antibody and MERTK knockdown in sh‐PROS1 TPC1 or K1 cells (**Figure** **6**A–C). Subcutaneous implantation in mouse models demonstrated that tumors injected with a 1:1 mixture of sh‐PROS1 K1 cells and PROS1‐overexpressing CAFs exhibited significantly larger volumes compared to the NC group. However, MERTK knockdown in sh‐PROS1 K1 cells reduced the tumor growth induced by PROS1‐overexpressing CAFs (Figure 6D).

To explore the potential autocrine effect of the PROS1‐MERTK interaction, PROS1 was overexpressed in PTC cells. The results revealed that PROS1 overexpression promoted migration, invasion, and proliferation, while anti‐PROS1 treatment or MERTK knockdown alleviated the PROS1‐induced migration and invasion of PTC cells (Figure S5A–C). In vivo subcutaneous implantation also demonstrated that tumors injected with PROS1‐overexpressing K1 cells were significantly larger than those in the NC group, with MERTK knockdown in K1 cells reducing the tumor growth driven by PROS1 overexpression (Figure S5D). These results indicate that the PROS1‐MERTK interaction facilitates tumor progression in PTC through both paracrine and autocrine mechanisms.

### 2.7 NFYB, FOXP2 Transcriptional Activation of PROS1 and Promotes PTC Progression Through the MERTK /WNT/TGF‐β Pathway

**Figure 7:**
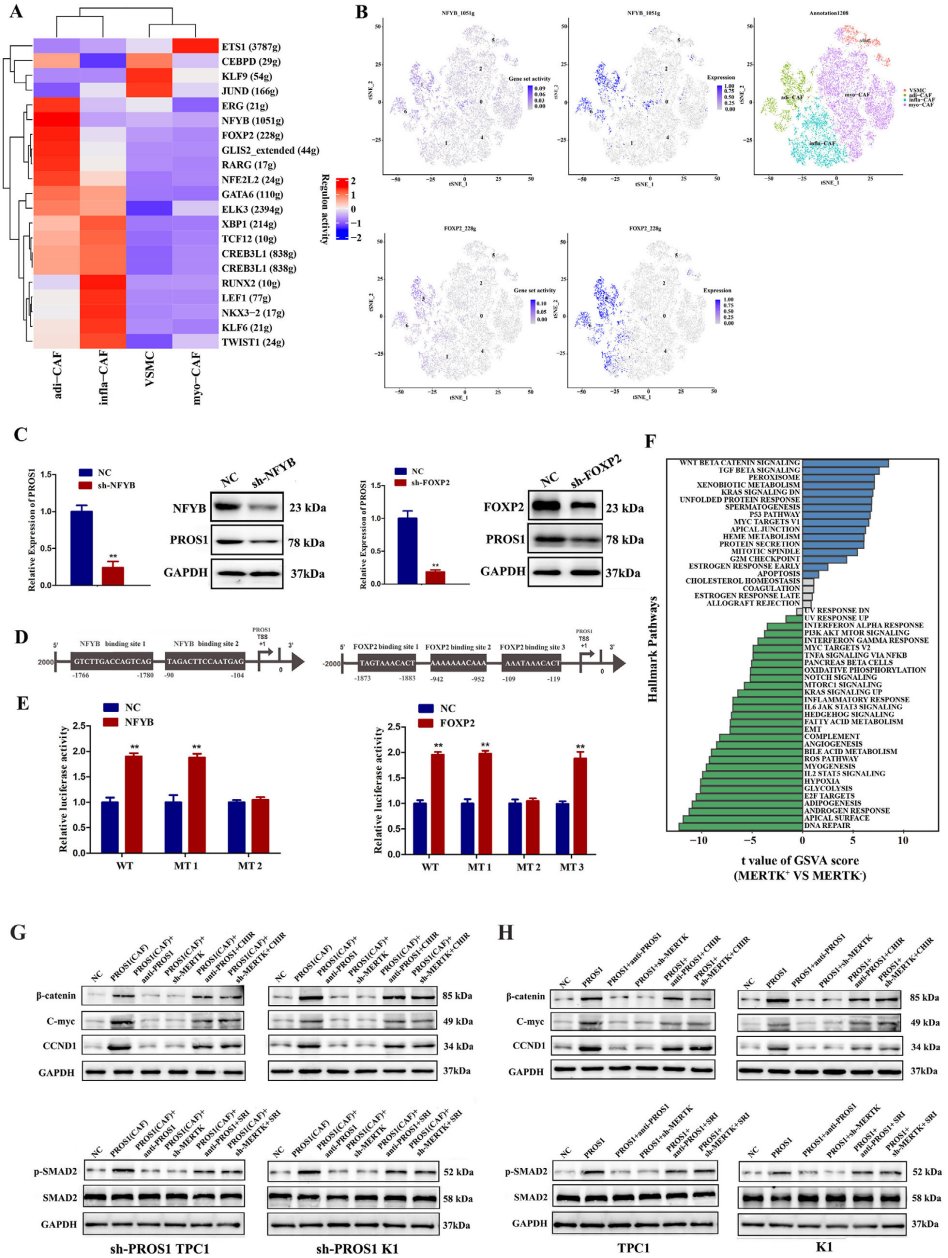
NFYB, FOXP2 transcriptional activation of PROS1 and promotion of PTC progression through the MERTK/WNT/TGF‐β pathway. A‐B) TF enrichment heatmaps and t‐SNE plots showing overexpression of FOXP2 and NFYB in adi‐CAFs, consistent with PROS1 expression. C) Knockdown of FOXP2 and NFYB significantly reduced PROS1 mRNA and protein expression (*n =* 3). D‐E) Dual luciferase reporter assays confirm that FOXP2 and NFYB bind to the PROS1 promoter (*n =* 3). F) GSEA of tumor cell data with high and low MERTK expression across 21 single‐cell transcription samples. G‐H) Western blot analysis verifying that PROS1 modulates the MERTK downstream mechanism through both paracrine and autocrine pathways (*n =* 3). All *P*‐values were determined using an unpaired two‐sided Student's t‐test. Data are presented as mean ± SD. **, *P* < 0.01.

To identify the upstream regulators of PROS1 in adi‐CAFs, gene co‐expression analysis was conducted, and each cell was scored for the activity of gene modules co‐expressed with and enriched for TF cis‐regulatory motifs using SCENIC analysis. Annotated target genes were categorized into high‐confidence and low‐confidence annotations. FOXP2 and NFYB were identified as high‐confidence transcriptional regulators of PROS1. tSNE and TF enrichment heatmaps demonstrated that adi‐CAFs overexpressed both FOXP2 and NFYB, in alignment with PROS1 expression (**Figure** **7**A,B). Knockdown of FOXP2 and NFYB in CAFs significantly decreased PROS1 mRNA and protein expression (Figure 7C). Dual luciferase reporter assays confirmed that FOXP2 and NFYB bind to the PROS1 promoter (Figure 7D,E). These results indicate that FOXP2 and NFYB transcriptionally activate PROS1 in CAFs. To explore the potential downstream mechanisms of MERTK, GSEA analysis was performed on tumor cell data with high and low MERTK expression from 21 single‐cell transcription samples. The results revealed significant activation of the WNT/β‐Catenin and TGF‐β signaling pathways (Figure 7F). Western blot analysis further confirmed that PROS1 influences the MERTK downstream signaling through paracrine or autocrine pathways (Figure 7G,H). Conditional media from CAFs overexpressing PROS1 significantly upregulated the expression of β‐catenin, C‐myc, CCND1, and p‐SMAD2 in thyroid cancer cells. However, PROS1 neutralizing antibody or MERTK knockdown in K1 or TPC1 cells inhibited the expression of these proteins (Figure 7G). Activation of WNT or TGF‐β pathways using activators (CHIR‐99021 and SRI‐011381) rescued the effects of PROS1 neutralization or MERTK knockdown (Figure 7G). Additionally, PROS1 overexpression in PTC cells enhanced β‐catenin, C‐myc, CCND1, and p‐SMAD2 expression, while anti‐PROS1 or sh‐MERTK reduced their expression (Figure 7H). WNT or TGF‐β pathway activators (CHIR‐99021 and SRI‐011381) effectively rescued the effects induced by PROS1 neutralizing antibody or MERTK knockdown (Figure 7H). These results demonstrate that PROS1 promotes PTC progression through the MERTK/WNT/TGF‐β pathway.

## 3 Discussion

The incidence of PTC has steadily risen over the years, with PTMC now accounting for more than 50% of cases.^[^ ^1^ ^]^ However, the mortality rate associated with PTMC has remained relatively stable. Many PTMC cases are subclinical and rarely progress to clinically significant thyroid cancer, allowing patients to live with the condition without intervention. For such cases, AS has been proposed as a treatment strategy.^[^ ^30^ ^]^ On the other hand, some PTMC cases exhibit invasive features, including central and lateral cervical LNM, microscopic extrathyroid invasion, gross extrathyroid invasion, lymphovascular invasion, and distant metastasis.^[^ ^31^ ^]^ These patients require comprehensive and standardized treatments, including surgical interventions, postoperative thyroid‐stimulating hormone inhibition therapy, and iodine‐131 therapy.^[^ ^30^ ^]^ As a result, accurately identifying high‐risk PTMC cases has become a key focus in clinical research.

Tumors form unique and intricate ecosystems, characterized by heterogeneous cell subpopulations with distinct molecular profiles, levels of aggressiveness, and proliferation potential, which interact to drive tumor progression. Understanding how this heterogeneity influences tumor dynamics holds important clinical value, particularly in improving diagnostic accuracy, prognostication, and predicting treatment responses. scRNA‐Seq technology enables the analysis of individual cells, thereby avoiding the signal‐averaging errors inherent in traditional genomic approaches.^[^ ^32^, ^33^ ^]^ Wang et al.’s single‐cell sequencing study of normal thyroid tissues, PTC, metastatic lymph nodes, and subcutaneous metastatic foci identified three distinct phenotypes of malignant thyrocytes‐follicular‐like, partial epithelial‐mesenchymal transition (EMT)‐like, and dedifferentiation‐like that shape the molecular subtypes, tumor characteristics, and radioiodine (RAI) responses of PTC. Prior research has identified multiple tumor subpopulation clusters that dominate as PTC progresses, each with distinct transcriptional profiles and evolutionary trajectories, which partly explain the limited efficacy of targeted therapies. Additionally, a study by Kolonin et al. identified a cluster of adi‐CAFs derived from adipocyte precursor cells, which secrete neuregulin 1 and contribute to drug resistance in urothelial carcinoma.^[^ ^34^ ^]^ In the present study, adi‐CAFs were found to upregulate pathways associated with cancer progression, such as angiogenesis. As the tumor progresses, adi‐CAFs infiltration increases, forming a nearly complete barrier around the tumor, preventing immune cells from reaching and attacking the cancer. scRNA‐Seq and spatial transcriptomics investigated the cellular components of PTC at different stages. Our findings suggest that changes in the local tumor microenvironment trigger cancer cell transitions from a primary state to a more advanced stage. Progression of PTC requires or is at least facilitated by, supportive immunity and extracellular matrix remodeling. Notably, specific upregulation of PROS1‐MERTK‐mediated cell‐cell communication was identified in progressive PTMC and advanced PTC tissues.

TAM receptors, which function as carcinogenic receptors on tumor cells and immunosuppressive receptors on immune cells, play pivotal roles in tumor progression and immune evasion, marking a new frontier in cancer biology.^[^ ^35^, ^36^, ^37^, ^38^ ^]^ By screening signaling pathways in the CellChat database based on the progression levels in PTC (normal < stage I < stage II ≤ stage III), the ligands PROS1 and the MERTK receptor in the TAM pathway were identified as closely associated with PTC progression. Single‐cell data indicate that PROS1, a key TAM signal, is specifically expressed in tumor epithelial cells, CAFs, and endothelial cells from progressive tumors, while its expression is minimal in normal thyroid tissues. MERTK, on the other hand, is predominantly expressed in myeloid cells and tumor epithelial cells of progressive PTMC and PTC, with little to no expression in normal tissues and non‐progressive PTMC. Notably, PROS1 expression in fibroblasts strongly overlaps with adi‐CAFs, suggesting that adi‐CAFs may contribute to tumor progression through PROS1 secretion. Additionally, MERTK expression in epithelial cells overlaps with several tumor epithelial cell clusters, indicating that progressive PTC is more likely to receive signals from the upstream TAM pathway due to elevated MERTK expression. Previous research has highlighted the critical roles of PROS1‐MERTK ligand‐receptor interactions in the development of various cancers. For example, PROS1 secreted by melanoma, breast cancer, and prostate cancer cells binds to MERTK and TYRO3 receptors on macrophages, inhibiting M1 polarization and supporting tumor growth, thereby promoting tumor progression.^[^ ^39^ ^]^ Myeloid‐derived PROS1 also alleviates peripheral inflammation and suppresses lung cancer invasion and metastasis.^[^ ^40^ ^]^ Multiple immunofluorescence assays, Western blot analyses, and dot blot assays confirmed the expression of PROS1 and MERTK in CAFs and PTC tumor cells. PROS1 from CAFs and tumor cells facilitated the migration and invasion of thyroid cancer cells expressing MERTK, emphasizing the significance of the PROS1/MERTK signaling pathway in PTC progression both in vitro and in vivo. These results are consistent with previous studies showing that AXL and PROS1 expression in PTC tissues correlates with resistance to radioactive iodine therapy, disease recurrence, and poor prognosis.^[^ ^27^, ^41^, ^42^ ^]^ Furthermore, tyrosine kinase inhibitor‐based targeted therapies have shown potential in treating metastatic thyroid cancer, including refractory PTC resistant to radioactive iodine.^[^ ^43^, ^44^, ^45^ ^]^

Although numerous hospitals worldwide have implemented active surveillance for PTMC as an alternative to immediate surgery, identifying which PTMC cases will clinically progress and necessitate early intervention remains challenging. Previous reviews have identified various molecular alterations‐such as gene mutations, mRNAs, noncoding RNAs, proteomics, and immune cell profiles potential biomarkers linked to PTMC progression. However, no single factor has proven sufficient for accurately predicting PTMC progression. The PROS1/MERTK pathway plays a key role in PTC progression, with single‐cell data revealing that MERTK is infrequently expressed in normal thyroid follicular epithelium and non‐progressive PTMC epithelial cells, but is significantly upregulated in progressive PTMC and PTC. Consequently, determining whether MERTK expression can serve as a marker for tumor progression is a central focus of this investigation. Expanding the sample size and performing additional immunohistochemistry across various PTC stages confirmed that MERTK expression in tumor cells aligns with trends observed in single‐cell data, suggesting that MERTK protein could serve as a biomarker for early diagnosis of PTMC progression. MERTK inhibitors have demonstrated the ability to promote tumor cell apoptosis and enhance the efficacy of immunotherapy in several cancer models. For example, the MERTK inhibitor Mer590 reduced cell‐surface MERTK levels by 87%, decreased colony formation, increased apoptosis, and enhanced chemosensitivity to carboplatin in non‐small cell lung cancer.^[^ ^46^ ^]^ The dual AXL/MERTK inhibitor INCB081776 induces a proinflammatory tumor immune microenvironment and enhances the effectiveness of anti‐PD‐L1 therapy in head and neck cancer.^[^ ^47^ ^]^ Sitravatinib, another MERTK inhibitor, sensitizes resistant hepatocellular carcinoma to anti‐PD‐L1 therapy by promoting tumor ferroptosis and reducing the infiltration of myeloid‐derived suppressor cells into the tumor microenvironment.^[^ ^38^ ^]^ These findings suggest that MERTK holds potential as a therapeutic target for the advanced progression of PTMC.

The mechanisms underlying PROS1 activation in adi‐CAFs and MERTK activation in tumor cells were also investigated. Activation of the transcription factors NFYB and FOXP2 was found to significantly upregulate PROS1 expression in adi‐CAFs, while MERTK further promotes PTMC progression through modulation of the WNT/β‐catenin and TGF‐β pathways. Previous studies have shown that the regulation of NFYB is primarily driven by other transcription factors. For instance, NFYB can be activated by E2F1 in osteosarcoma and E2F4 in hepatocellular carcinoma.^[^ ^48^, ^49^ ^]^ FOXP2 is primarily regulated by various noncoding RNAs, including let‐7b, miR‐34a, miR‐762, SNHG1, and miR‐23b, in breast cancer, glioma, and colon cancer.^[^ ^50^, ^51^, ^52^ ^]^ Furthermore, recent work by Yu et al. demonstrated that ALKBH5 can regulate m6A demethylation in FOXP2 mRNA, facilitating cell cycle entry and EMT in ovarian cancer.^[^ ^53^ ^]^

In conclusion, this study highlights the PROS1/MERTK signaling axis as a critical component in the cellular microenvironment driving PTMC progression. These findings provide new insights and potential strategies for targeted interventions in patients with high‐risk PTMC.

## 4 Experimental Section

### Patient Selection

Nineteen samples were obtained from fifteen patients who underwent surgery at the Department of Thyroid Surgery, The First Hospital of China Medical University. These included four para‐tumoral (normal) tissues, four non‐progressive PTMC (stage I) tissues, five progressive PTMC (stage II) tissues, and six progressive PTC (stage III) tissues. All included PTCs or PTMCs were excluded from autoimmune thyroiditis and thyroid dysfunction. Non‐progressive PTMC was defined as cases under active surveillance for five years post‐initial diagnosis without clinical progression. Tumors exhibiting clinical progression, such as tumor enlargement, LNM, or ETE, were classified as progressive PTMCs. Spatial transcriptomics sequencing was performed on paraffin‐embedded sections from six patients (2 non‐progressive PTMCs, 2 progressive PTMCs, and 2 progressive PTCs). Hematoxylin and eosin‐stained sections were reviewed by two experienced pathologists to confirm pathological findings, with clinical data detailed in Table S1 (Supplementary Information). All procedures involving human subjects adhered to ethical standards set by the Research Ethics Committee of The First Hospital of China Medical University (AF‐SOP‐07‐1.1‐01).

### Sample Preparation For Single‐Cell Profiling

Biopsy tissues, with a minimal size of 5 mm^3^, were digested using a digestion solution containing DNase I (Sigma), 0.2% collagenase I/II (Thermo Fisher Scientific), and 25 units of dispase (Invitrogen) in Dulbecco's modified Eagle's medium (DMEM, Thermo Fisher Scientific) within 2 h to generate a single‐cell suspension. The concentration of viable cells was determined using an immunofluorescence‐based automated cell counter (Luna FL Cell Counter, Logos Biosystems). Cells were maintained on ice throughout the process to prevent dissociation‐related artifacts.

### Droplet‐based scRNA‐Seq

Single‐cell suspensions were loaded onto the 10x Chromium platform to capture 5000 single cells, following the 10x Genomics Chromium Single‐Cell 3′ kit (V3) protocol. cDNA amplification and library construction were carried out as per the standard procedure. Libraries were sequenced on an Illumina NovaSeq 6000 sequencing system (paired‐end multiplexing run, 150 bp) by LC‐Bio Technology Co. Ltd. (Hangzhou, China), with a minimum depth of 20000 reads per cell.

### Quality Control and Batch Effect Mitigation in scRNA‐Seq Data Analysis

The “Seurat” R package (version 4.2.0) was employed as the central bioinformatics tool. Cells were excluded if they met any of the following criteria: fewer than 200 unique molecular identifiers (UMIs), expression of over 6000 or under 2001 genes, or if more than 20% of their UMIs were derived from the mitochondrial genome. Doublets were identified and removed using the “DoubletFinder” package (version 2.4) with default settings. The “Log‐Normalize” method, a global‐scaling normalization technique, was used to normalize feature expression for each cell by considering total expression and applying a scaling factor of 10000. The normalized values were then log‐transformed using Seurat's “NormalizeData()” function. After normalization, the normalized expression profiles from all samples were merged into a unified dataset using R's “merge()” function (version 4.1.3). To correct for batch effects, the “harmony” package (version 0.1.0) was used. The top 5000 highly variable genes (HVGs) within the merged dataset were identified using the “FindVariableFeatures()” function.

### Unsupervised Clustering and Dimensionality Reduction For Pattern Discovery

Top principal components were calculated using the “PCElbowPlot()” function from Seurat (version 4.2.0) based on the refined gene expression profiles of the top 5000 HVGs after batch effect correction. Subsequently, cell clustering was performed using Seurat's “FindNeighbors()” and “FindClusters()” functions. Major cell types were identified by their distinct expression patterns of preselected marker genes, including *CSF1R*, *FCER1G*, and *LYZ* for myeloid cells; *CD3D*, IL7R, and *NKG7* for T and NK cells; *CD79A*, *MS4A1*, *IGKC*, and *JCHAIN* for B cells/plasma cells; *TG*, *EPCAM*, and *KRT19* for thyroid epithelial cells; *RGS5*, *DCN*, *COL1A1*, and *ACTA2* for fibroblasts; and *RAMP2*, *VWF*, and *FLT1* for endothelial cells. An iterative “high‐resolution” approach was employed to define finer subclusters within each major cell type, enhancing our understanding of cellular heterogeneity.

### Identification of Distinctive Genes in Cell Clusters

To identify signature genes for each subcluster, the “FindAllMarkers()” function in Seurat was used to detect differentially expressed genes (DEGs), with significance determined via the Wilcoxon rank‐sum test and corrected for multiple comparisons using Bonferroni correction.

### Pathway Enrichment Analysis

Gene Ontology (GO) and Kyoto Encyclopedia of Genes and Genomes (KEGG) analyses were conducted to investigate the biological functions and relevant signaling pathways associated with each cell type, using the “clusterProfiler” R package. These analyses specifically focused on biological process categories in GO and pathways with adjusted *P*‐values < 0.05 were considered significantly enriched. To further investigate cellular functions, Gene Set Variation Analysis (GSVA) was performed using hallmark pathway sets.

### Pseudotime Trajectory Analysis

Pseudotime trajectory analysis was conducted using the Monocle 2 algorithm, allowed to map the differentiation and transition of cell subtypes. For this, the Seurat v3.1.2 function “FindVariableFeatures” was used to select the top eight genes with the highest variation from each cluster to construct the trajectory.

### Cellular Communication Analysis

Cell‐cell communication between immune/stromal cells and cancer cells was analyzed using CellPhoneDB (version 2.1.0) based on known ligand‐receptor pairings. The permutation number was set to 1000 to establish the null distribution of the average expression of ligand‐receptor pairs across randomized cell identities. A threshold for receptor or ligand expression was applied across each cell type, based on the average log‐transformed gene expression distribution. Interaction pairs were considered significant if the average log‐transformed expression exceeded 0.1 and the *P*‐value was < 0.05.

### SCENIC Analysis

To analyze the activated regulons in adi‐CAFs, standard SCENIC procedures were employed.^[^ ^54^ ^]^ A co‐expression network of transcription factors (TFs) and gene sets was constructed using the runGenie package, and the RcisTarget package was used to identify potential direct binding targets of the transcription factors. Finally, the AUCell package was used to calculate regulon activity scores for each cell. Statistical significance of regulon expression in CAFs between groups was evaluated using the Wilcoxon test, with *P*‐values corrected using the Holm‐Bonferroni method.

### Spatial Transcriptomics Sequencing

Following resection, tissue samples were collected and subjected to a tissue optimization experiment using the 10× Genomics Visium Spatial Tissue Optimization (Rev A) platform. Initially, tissue staining and imaging protocols were carried out, followed by tissue permeabilization and fluorescent cDNA synthesis, with RNA quality assessed (RIN > 7.0). After tissue removal, slide imaging was conducted, and Visium Spatial Gene Expression analysis was performed using the 10× Genomics Visium Spatial Gene‐Expression Reagent Kits (Rev B). Tissues were first fixed and stained for imaging, followed by tissue permeabilization at various durations (3, 6, 12, 18, 24, and 30 min). Notably, a 12 min permeabilization period yielded the highest fluorescence signal in both tumor and adjacent normal thyroid regions. This step was followed by reverse transcription, second‐strand synthesis, denaturation, cDNA amplification, quality control, Visium spatial gene‐expression library construction, and ST sequencing according to the manufacturer's instructions. Subsequent analyses adhered to the standard procedures for single‐cell sequencing.

### Isolation of Primary Cells

Primary normal epithelial cells, tumor cells, normal fibroblasts (NFs), and CAFs were isolated from fresh normal thyroid, non‐progressive PTMC, progressive PTMC, and progressive PTC tissues. Tumor tissues were sectioned into 1 mm^3^ pieces using a razor blade and dissociated with the Tumor Dissociation Kit (Miltenyi Biotec) per the manufacturer's guidelines. The resulting cell suspension was filtered through a 100 µm cell strainer, resuspended, and cultured in a T75 flask. Epithelial cells and fibroblasts, which are both adherent cell types, were separated through differential centrifugation, exploiting their distinct adherence and digestion properties. Primary NFs and CAFs were characterized by immunofluorescence with positive α‐SMA staining, while primary tumor cells were characterized by positive EPCAM staining.

### Culture of Primary Cells and Cell Lines

The K1 cell line was obtained from the European Collection of Authenticated Cell Culture (UK), and the TPC1 cell line was kindly provided by Professor Meiping Shen from the Department of General Surgery at the First Affiliated Hospital of Nanjing Medical University, Nanjing, Jiangsu. K1 cells were cultured in a medium consisting of DMEM, Ham's F12, and MCDB 105 (2:1:1 ratio), supplemented with 2 mm glutamine and 10% fetal bovine serum (FBS). TPC1 cells and primary tumor cells were cultured in DMEM with 10% FBS. Commercial primary CAFs from thyroid cancer were purchased from MEISEN CELL (Zhejiang, China), while primary fibroblast cells were cultured in DMEM/F12 medium (Gibco) supplemented with 10% FBS (Gibco) and 1% antibiotics (Invitrogen). Primary normal epithelial cells were cultured in Roswell Park Memorial Institute‐1640 medium supplemented with 10% FBS.

### Preparation of Small Interfering RNA (siRNA)

Expression plasmids and human shRNA were sourced from Obio Technology (Shanghai, China). Transfection of shRNAs or plasmids into cells was performed using Lipofectamine 3000 Reagents (Invitrogen, USA) according to the manufacturer's instructions. The shRNA sequences used were: sh‐PROS1: (Sense: 5′‐GCG UGA UAC UGU ACG CAG ATT‐3′), sh‐MERTK: (Sense: 5′‐GGA UGA AGC UCC GAC UAA TT‐3′), sh‐NFYB: (Sense: 5′‐CCG AUU GCA AAC GUG GCA ATT‐3′), and sh‐FOXP2: (Sense: 5′‐GCA GCA GAU CCU UCA GCA ATT‐3′).

### Dot Blot Assay

To collect the supernatant from the primary cell culture medium, 2 µL of the sample was evenly applied to a nitrocellulose membrane and air‐dried for 5–10 min. The membrane was then blocked with 5% non‐fat milk for 1 h, followed by overnight incubation at 4 °C with primary antibodies against GAS6 (1:1000, Cell Signaling Technology, Cat#67202) and PROS1 (1:1000, Abcam, Cat#ab280885). Protein dots were visualized via chemiluminescence (Thermo, USA) after incubation with secondary antibodies (1:5000, Cell Signaling Technology, Cat#14708).

### Multiplex Immunohistochemistry (mIHC) Staining Assay

For mIHC staining, formalin‐fixed, paraffin‐embedded tissue sections were processed using mIHC/immunofluorescence kits (Absin, Shanghai, China, Cat#abs50014) following the manufacturer's instructions. Primary antibodies included PROS1 (1:1000, Abcam, Cat#ab280885), GAS6 (1:100, Cell Signaling Technology, Cat#67202), ACTA2 (1:1000, Abcam, Cat#ab124964), CD31 (1:400, Abcam, Cat#ab76533), CD163 (1:500, Abcam, Cat#ab182422), MERTK (1:1000, Abcam, Cat#ab52968), and AXL (1:2000, Abcam, Cat#ab219651). Fluorescent signals were captured and merged using a laser scanning confocal microscope (ZEISS, LSM880) and inForm software (version 2.42).

### Immunohistochemistry and Western Blot Analysis

Fresh tumors and corresponding non‐cancerous tissues were collected from 50 non‐progressive PTMC (stage I), 50 progressive PTMC (stage II), 50 progressive PTC (stage III), and 50 paratumoral (normal) samples. IHC staining was evaluated using the semi‐quantitative Remmele scoring system, which classifies protein expression based on the percentage of positive cells and the staining intensity. Five random visual fields were selected from each section. Staining intensity was scored as 0 (no staining), 1 (low staining), 2 (intermediate staining), and 3 (high staining), while the staining area was scored as 0 for ≤ 5%, 1 for 5–25%, 2 for 26–50%, 3 for 51–75%, and 4 for > 75%. The histological score was calculated as the product of staining intensity and staining area, with scores classified as follows: 0 for negative (−), 1–4 for weakly positive (+), and 6–12 for strongly positive (++).^[^ ^55^ ^]^ For Western blot analysis, total proteins were extracted from the cells using a lysis buffer, fractionated by 10% or 12% SDS‐PAGE, and transferred to polyvinylidene fluoride membranes (Bio‐Rad, Hercules, CA, USA). Membranes were blocked with 5% skim milk for 2 h at room temperature, then incubated with primary antibodies overnight at 4 °C. The membranes were subsequently incubated with horseradish peroxidase‐conjugated anti‐rabbit immunoglobulin G (1:20 000) secondary antibody for 2 h at room temperature. Primary antibodies included TYRO3 (1:1000, Abcam, Cat#ab109231).

### Transwell, CCK8, and Colony Formation Assay

To examine the interaction between PROS1 secreted by CAFs and MERTK receptors in tumor cells, primary thyroid cancer CAFs (Meisen Cell, Zhejiang, China) and PTC cell lines were used. The experimental groups included NC (sh‐PROS1 PTC cells), supernatant of PROS1 overexpressing CAFs + sh‐PROS1 PTC cells, supernatant of PROS1 overexpressing CAFs + PROS1 neutral antibody + sh‐PROS1 PTC cells, and supernatant of PROS1 overexpressing CAFs + sh‐PROS1 and sh‐MERTK PTC cells. To verify tumor cells’ role in promoting PTC progression through autocrine PROS1 interaction with MERTK receptors, the groups were as follows: NC (PTC cells), PROS1 overexpressing PTC cells, PROS1 overexpressing PTC cells + PROS1 neutral antibody, and PROS1 overexpressing PTC cells + sh‐MERTK PTC cells. Detailed protocols for Transwell, CCK8, and colony formation assays are presented in the previously published articles.^[^ ^56^, ^57^ ^]^

### Tumor Xenograft Model

Xenograft tumor models were established in 4‐ to 5‐week‐old BALB/c nude mice sourced from Beijing Vital River Laboratory Animal Technology Co., Ltd. (Beijing, China). To investigate the interaction between PROS1 secreted by CAFs and MERTK receptors in tumor cells, primary thyroid cancer CAFs from Meisen Cell (Zhejiang, China) and a PTC cell line were used. The experimental groups included: NC (sh‐PROS1 PTC cells), PROS1 overexpression CAFs + sh‐PROS1 PTC cells, and PROS1 overexpression CAFs + sh‐PROS1 and sh‐MERTK PTC cells. To confirm that tumor cells promote PTC progression through the autocrine interaction between PROS1 and tumor cell MERTK receptors, the groups were as follows: NC (PTC cells), PROS1 overexpressing PTC cells, and PROS1 overexpressing + sh‐MERTK PTC cells. All animal studies adhered to the Institutional Animal Care and Use Committee (IACUC) guidelines of China Medical University (NO. KT20240659).

### Reporter Vector Construction and Luciferase Reporter Assay

To construct reporter vectors, the promoter regions of PROS1 were cloned and amplified as wild‐type (WT) vectors, while promoter regions with deleted NFYB and FOXP2 binding sequences were cloned and amplified as mutant‐type (MT) vectors. CAFs were seeded in 96‐well plates and co‐transfected with NFYB and FOXP2 overexpression vectors or empty vectors and whole or deleted promoter region vectors using Lipofectamine 3000. After 48 h, luciferase activity was measured using a dual luciferase reporter system (Promega, Beijing, China).

### Quantification and Statistical Analysis

Statistical analyses were performed using R (version 3.1.4) and GraphPad Prism 8.0. The assays were repeated at least three times, and data are presented as mean ± standard deviation. Student's t‐test, Wilcoxon rank‐sum test, and log‐rank test were employed for statistical analysis. *P*‐values < 0.05 were considered statistically significant (ns, *P* ≥ 0.05; *, *P* < 0.05; **, *P* < 0.01; ***, *P* < 0.001; ****, *P* < 0.0001).

## Conflict of Interest

The authors declare no conflict of interest.

## Author Contributions

W.‐Q.Z., Y.Z. and Z.L. contributed equally to this work, and they are co‐first authors. W.S., W.‐Q.Z., H.Z., and H.‐Q.W. designed this study and performed the statistical analysis, X.‐Y.J., Y.Z., and Z.‐Y.W. analyzed the data; H.‐Y.S., Z.L., and F.Y. provided clinical samples and prepared figures; L.‐R.Y. and Y.X. revised the manuscript; H.Z. and W.S. supervised the study. All authors read and approved the manuscript.

## Supporting information



Supporting Information

## Data Availability

The data that support the findings of this study are available from the corresponding author upon reasonable request.;
