# Supplementary material for: PROS1‐MERTK Axis Drives Tumor Microenvironment Crosstalk and Progression in Papillary Thyroid Microcarcinoma
Source: Adv Sci (Weinh). 2025 May 28;12(30):e13474. doi: 10.1002/advs.202413474 (PMC12376618; doi:10.1002/advs.202413474)
Supplement: Supplementary file 1 — Supporting Information [file ADVS-12-e13474-s001.docx]

**Supplementary data**

**Fig.S1** Quality control of the single-cell sequencing data. **A)**, **B)**, **C)** Mitochondrial content, number of genes, and counts before quality control. **D)**, **E)**, **F)** Mitochondrial content, number of genes, and counts after quality control. **G)** Bar plots indicating the proportion of 32 clusters in each sample. **H)** H&E staining and spatial distribution of different clusters for spots throughout the tumor (with adjacent) tissues in the 10x Visium capture slides. **I)** Map the 3 clusters of thyrocytes identified by single-cell sequencing onto the other slices. **J)** Heatmap showing the 50 significantly enriched hallmark pathways (rows) for thyrocytes of each cluster (columns).


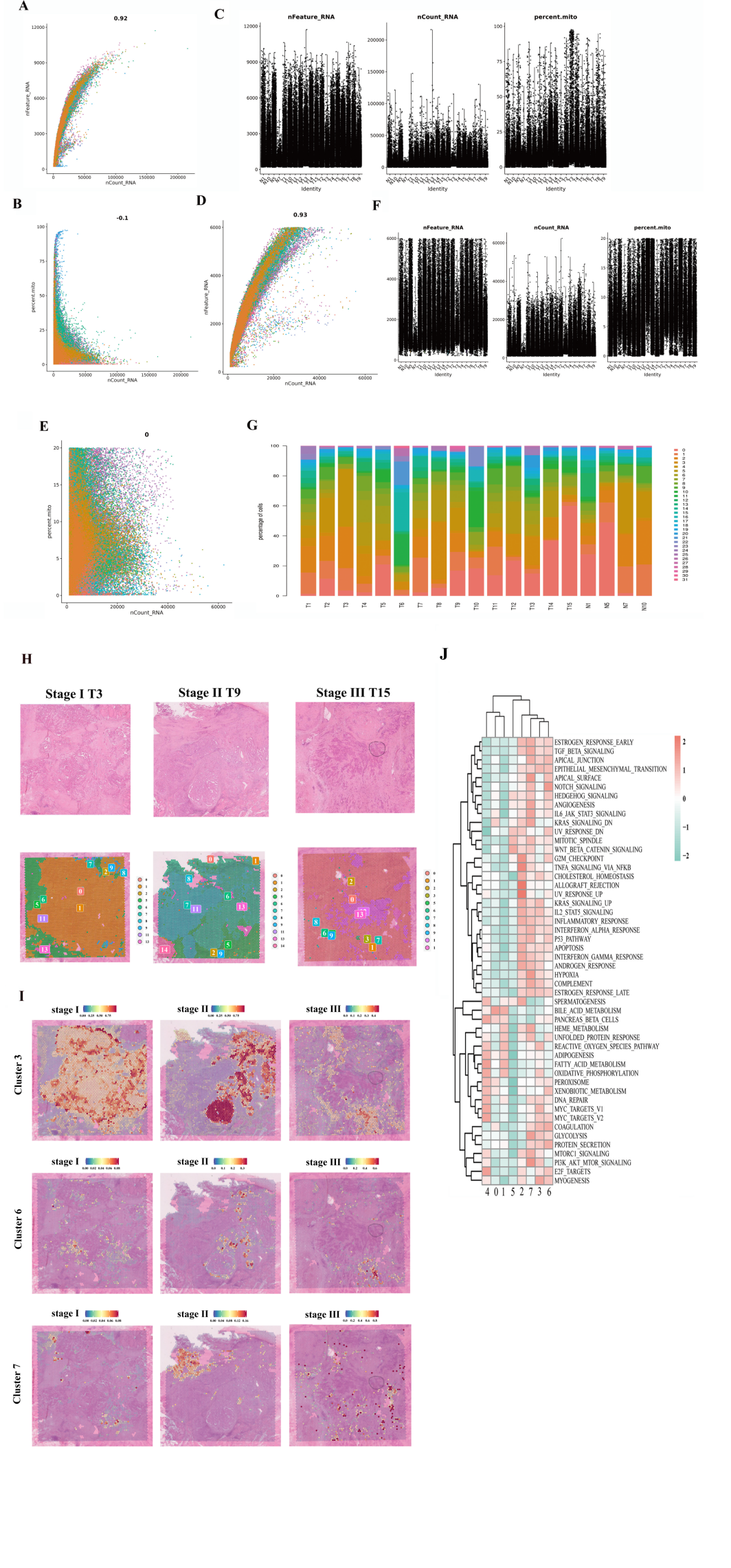


**Fig.S2 A-H)** KEGG enrichment of each thyrocyte subcluster. Circle plots showing the interaction between **I)** GAS6-AXL, **J)** PROS1-AXL, and GAS6-MERTK ligand-receptor pairs in the major cell types of different-stage tumors and normal thyroid. **K)** Circle plots showing the interaction between IFNG-IFNGR1+IFNGR2 ligand-receptor pair in the major cell types of different-stage tumors. **L)** Violin plots showing the expression of IFNG, IFNGR1, and IFNGR2 in each major cell type. **M)** Violin plots showing the expression of POSTN, ITGAV, ITGB3, and ITGB5 in each major cell type. **N)** Circle plots showing the interaction between POSTN-ITGAV+ITGB5 ligand-receptor pair in the major cell types of different-stage tumors. **O)** Violin plots showing the expression of PROS1 and MERTK gene in thyrocytes and PROS1 gene in fibroblasts. **P)** Violin plots showing the expression of MERTK gene in macrophages at different stages. **Q)** KEGG and GOBP enrichment of MERTK highly expressing macrophages. **R)** Expression of PROS1 and MERTK on the other spatial slices.


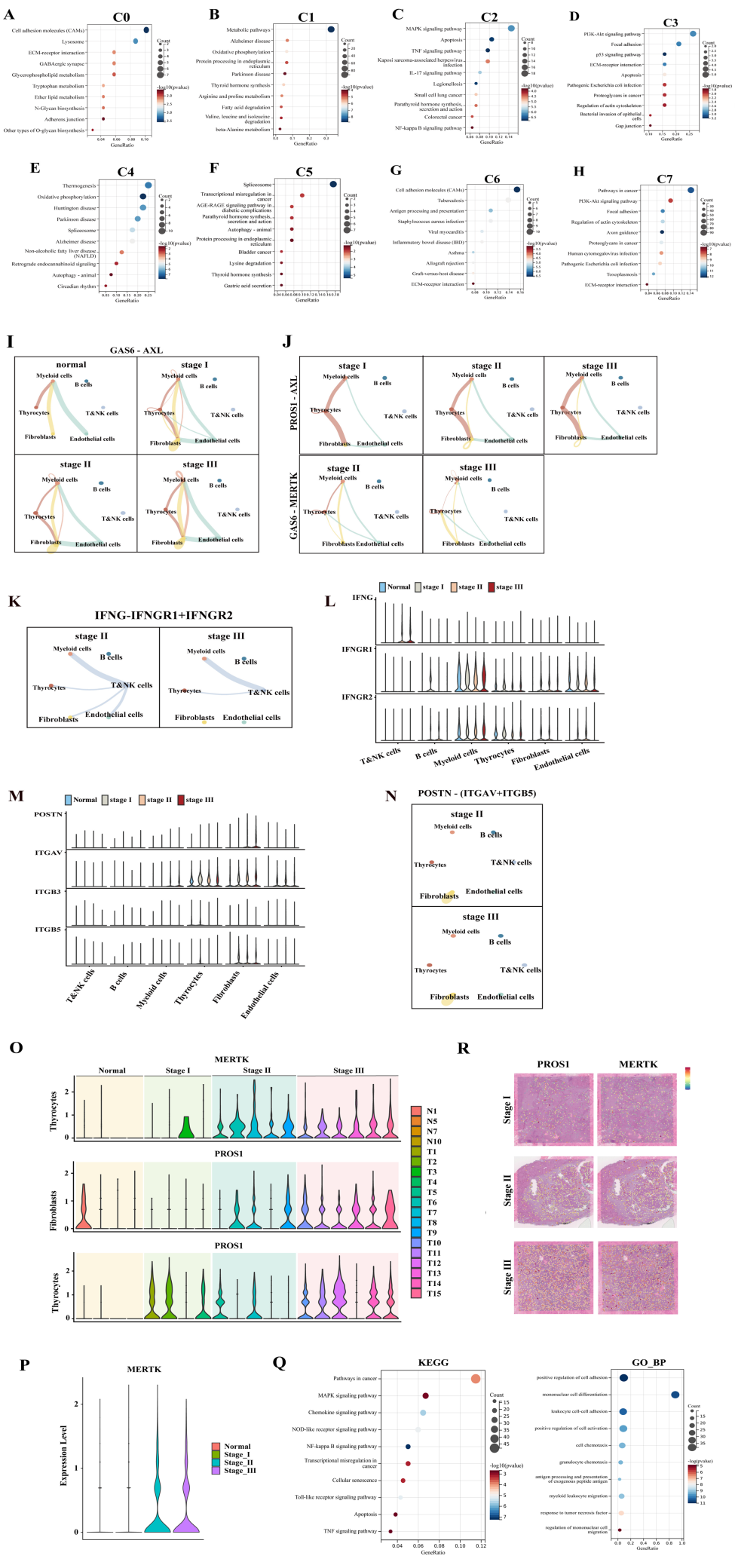


**Fig.S3** **A)** UMAP plot of the fibroblast landscape colored by subclusters. **B)** Dot plot showing the expression levels of the top 3 marker genes in each fibroblast subcluster. **C)** Dot plot showing the expression of PROS1 in each fibroblast subtype. **D)** Dot plot showing the expression of PROS1 in fibroblasts at each stage. Pseudotime trajectory analysis of fibroblasts grouped by state **E)** and stage **F)**. Mapping of the infla-CAFs **G)** and myo-CAFs **H)** identified by single-cell sequencing onto the slices. **I)** Mapping of the adi-CAFs identified by single-cell sequencing onto the other slices.


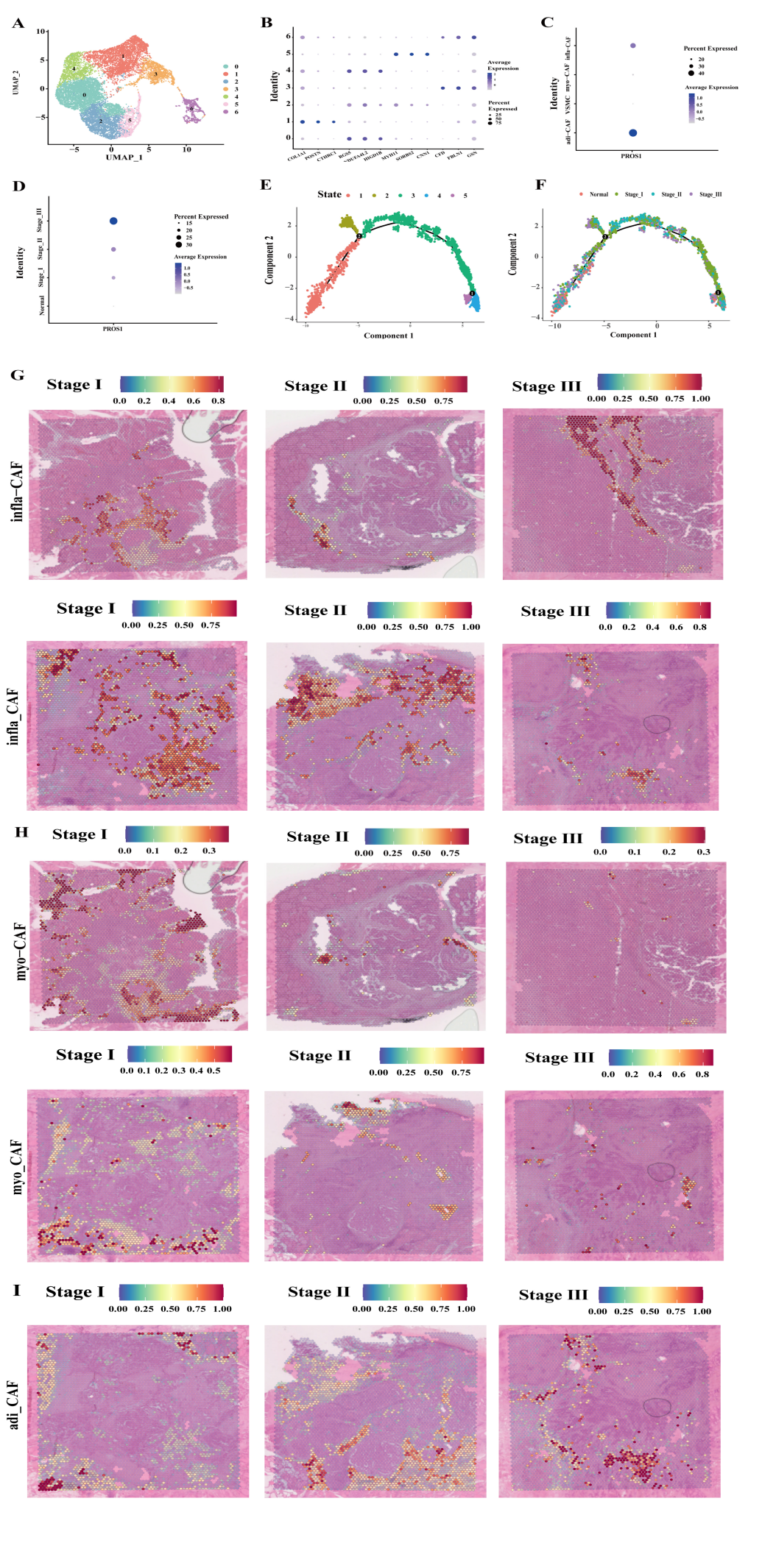


**Fig.S4** **A)** IF staining of GAS6 and AXL obtained from normal thyroid tissues and PTC samples at different stages (n=10 per group). **B)** Expression difference among non-cancerous tissues (n=50) and cancer tissues in three stages (n=50 per group). **C)** Western blotting analysis showing the protein expression levels of TYRO3 gene in primary epithelial cells derived from four types of tissues (n=3). **D)** Dot blot assay showing the expression of GAS6 in the supernatant of primary fibroblast culture medium (n=3). **E)** Feature plot showing the expression of MERTK in thyrocytes. **F)** Significant overexpression of PROS1 in tumor confirmed using the TCGA database. *P*-value was determined using an unpaired two-sided Wilcoxon rank-sum test. *, *P* < 0.05, ***, *P* < 0.001.


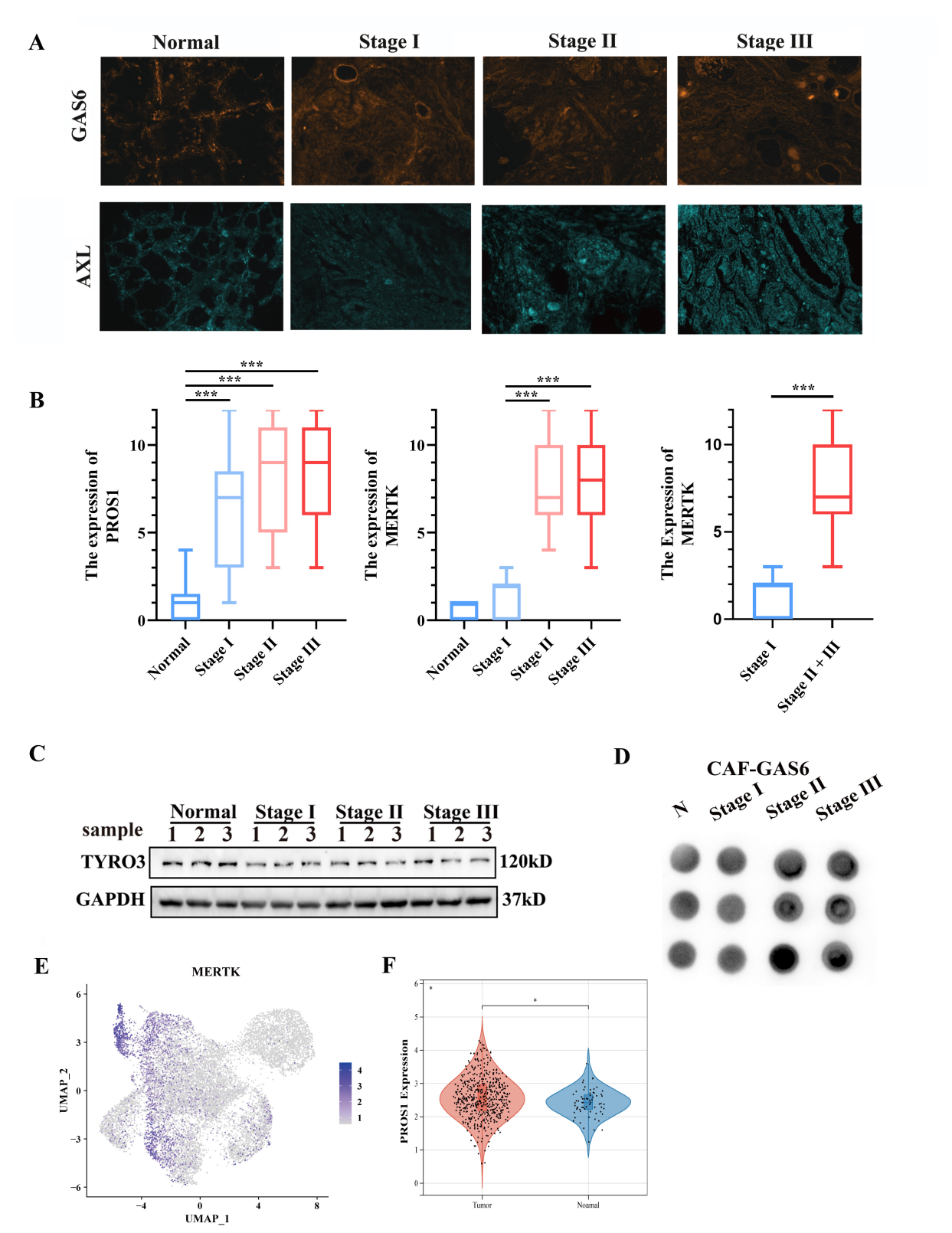


**Fig.S5** **A)** Transwell assays used to evaluate the migration and invasion in PTC cells with various treatments targeting autocrine (n=3). **B)** Proliferation of PTC cells with various treatments targeting autocrine evaluated by CCK-8 assay (n=3). **C)** Colony formation assay used to evaluate the proliferation ability of PTC cells after various treatments targeting autocrine (n=3). **D)** Subcutaneous implant mouse models inoculated with NC, Lv-PROS1 K1, or Lv-PROS1+sh-MERTK K1 cells (n = 5) and volumes of harvested tumors in each group. All *P*-values were determined using an unpaired two-sided Student’s t-test. Data presented as the mean ± SD. **, *P* < 0.01.


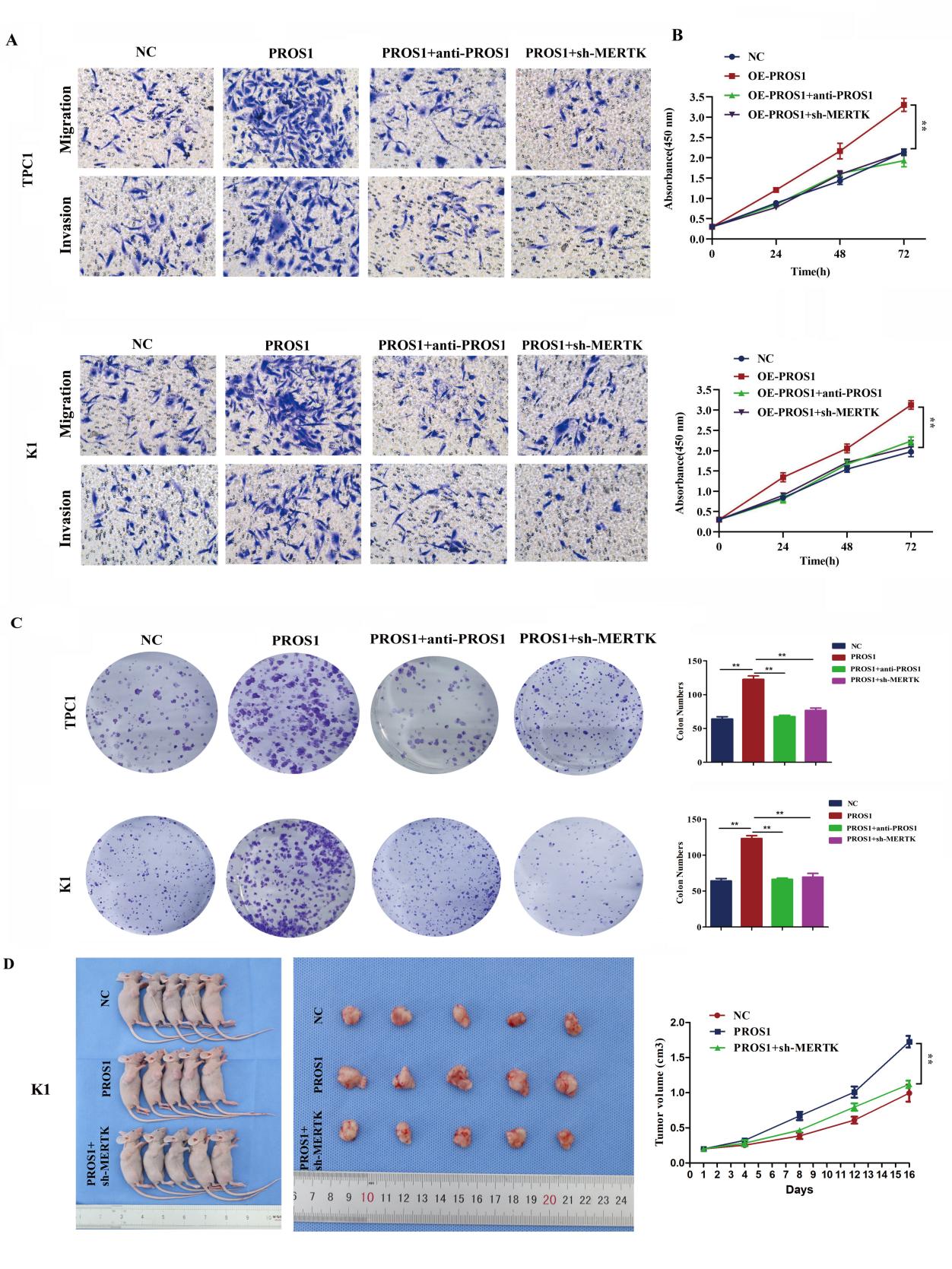


**Supplementary Table 1**

| Sample_ID | Grade | Age | Gender | TNM (AJCC 8th) | Extrathyroidal_extension | Distant_metastasis | Tumor_diameter (cm) | Histologic_subtype |
| --- | --- | --- | --- | --- | --- | --- | --- | --- |
| T1, N1 | Stage I | 48 | Female | T1aN0M0 | No | No | 0.84 | Classical |
| T2 | Stage I | 38 | Female | T1aN0M0 | No | No | 0.94 | Classical |
| T3 | Stage I | 65 | Male | T1aN0M0 | No | No | 0.93 | Classical |
| T4 | Stage I | 42 | Female | T1aN0M0 | No | No | 0.85 | Classical |
| T5, N5 | Stage II | 55 | Female | T1bN1aM0 | No | No | 1.30 | Classical |
| T6 | Stage II | 56 | Female | T1bN0M0 | No | No | 1.11 | Classical |
| T7,N7 | Stage II | 59 | Female | T1aN1bM0 | No | No | 0.93 | Classical |
| T8 | Stage II | 51 | Male | T1bN1bM0 | No | No | 1.36 | Classical |
| T9 | Stage II | 56 | Female | T4aN1aM0 | Left recurrent laryngeal nerve | No | 0.72 | Classical |
| T10,N10 | Stage III | 34 | Female | T3bN1bM1 | Strap muscles | Pulmonary metastasis | 1.94 | Diffuse Sclerosing Variant |
| T11 | Stage III | 65 | Male | T4aN1aM0 | Larynx (cricoid cartilage) | No | 2.10 | Classical |
| T12 | Stage III | 50 | Female | T4aN1bM1 | Hypopharyngeal constrictor muscle and esophagus | Pulmonary metastasis | 3.00 | Classical |
| T13 | Stage III | 49 | Male | T3bN1aM0 | Strap muscles | No | 4.48 | Classical |
| T14 | Stage III | 48 | Male | T4aN1bM0 | Left recurrent laryngeal nerve | No | 2.24 | Follicular  variant |
| T15 | Stage III | 32 | Male | T4aN1bM1 | Bilateral recurrent laryngeal nerves | Pulmonary metastasis | 4.00 | Follicular  variant |
